# Decryption of Active Constituents and Action Mechanism of the Traditional Uighur Prescription (BXXTR) Alleviating IMQ-Induced Psoriasis-Like Skin Inflammation in BALB/c Mice

**DOI:** 10.3390/ijms19071822

**Published:** 2018-06-21

**Authors:** Xiaobo Pang, Ke Zhang, Jian Huang, Hangyu Wang, Le Gao, Tao Wang, Yingnan Sun, Long Chen, Jinhui Wang

**Affiliations:** 1 School of Traditional Chinese Materia Medica, Shenyang Pharmaceutical University, Shenyang 110016, China; pangxiaobo126@126.com (X.P.); 13504051049@163.com (J.H.); 13940164780@163.com (L.G.); WangTao9492@163.com (T.W.); 17614665295@163.com (Y.S.); 2 School of Pharmacy, Shihezi University (Key Laboratory of Xinjiang Phytomedicine Resource and Utilization, Ministry of Education), Shihezi 832001, China; xjzk1984@163.com (K.Z.); 18909932852@189.com (H.W.); xiaochenlong2@126.com (L.C.); 3 Department of Medicinal Chemistry and Natural Medicine Chemistry (State-Province Key Laboratories of Biomedicine-Pharmaceutics of China), Harbin Medical University, Harbin 150081, China

**Keywords:** BXXTR, psoriasis, an-inflammation, active constituent, mechanism

## Abstract

Bai Xuan Xia Ta Re Pian (BXXTR) is a traditional Uighur medicine ancient prescription in China widely used in the treatment of psoriasis, presenting a high curative rate and few side effects. Given that the active constituents and action mechanism still remain unclear, the aim of this study is to explore the potential active constituents and mechanism of antipsoriasis of BXXTR. Psoriasis-like lesions model in BALB/c mice was induced by Imiquimod (IMQ), including five treatment groups: control group, IMQ-treated group, IMQ-ACITRETIN group (Positive control group), IMQ-BXXTR low dose group, IMQ-BXXTR medium dose group and IMQ-BXXTR high dose group. The Psoriasis Area and Severity Index (PASI) score, skin and ear thickness, and histologic section were collected. The differentially expressed genes were determined by using RNAseq technology and the relevant pathways were analyzed by KEGG database. The ELISA kit and western blot assays were used to detect the related protein expression levels. In addition, the chemical constituents of BXXTR were determined by UPLC-TOF-MS analysis and the potential active constituents were predicted by SEA DOCK and Gene Ontology (GO). The data demonstrated that BXXTR significantly alleviated IMQ-induced psoriasis. RNA-seq analysis showed that BXXTR induced the expression levels of 31 genes; the KEGG analysis suggested that BXXTR could significantly change IL-17-related inflammatory pathways. The ELISA kit confirmed that the expression level of IL-17A protein was significantly reduced. 75 compounds of BXXTR were determined by UPLC-TOF-MS analysis, 11 of 75 compounds were identified as potential active compounds by similarity ensemble approach docking (SEA DOCK) and Gene Ontology (GO). BXXTR reduced the severity of skin lesions by inhibiting IL-17-related inflammatory pathways. The results indicated that BXXTR could suppress psoriasis inflammation by multiple-constituents-regulated multiple targets synergistically. Collectively, this study could provide important guidance for the elucidation of the active constituents and action mechanism of BXXTR for the treatment of psoriasis.

## 1. Introduction

Psoriasis is one of the most common immune-mediated chronic inflammatory skin disorders characterized by neovascularization, hyperproliferative keratinocytes, abnormal keratinocyte differentiation, generally accompanied by marked parakeratosis and extensive inflammatory cell infiltration, including T cells, neutrophils, and dendritic cells (DCs). Psoriasis can cause systemic damage, seriously affecting the quality of life of patients [1]. Previous studies have shown that the formation of neovascularization in psoriatic skin lesions provides nutritional support for epidermal keratinocyte hyperplasia while inflammatory cells enter the diseased tissue through the highly permeable endothelium. On the other hand, the infiltration of inflammatory cells accelerates the formation of neovascularization. Three factors form a positive feedback to each other, leading to repeated or even worsened the condition of psoriasis [2].

In recent years, a large number of studies have confirmed a new population of IL-17-producing Th cells involved in the autoimmune model system. IL-23, as a key initiation cytokine in autoimmune development, is associated with the development and maintenance of Th17 cells [3,4]. Elevated levels of IL-23 and Th17-associated cytokines in the serum and in cutaneous lesions of psoriatic patients indicate an important role of the IL-23/IL-17 Axis in the pathogenesis of psoriasis, Including IL-23, IL-17, IL-18, IL-22, IL-1β, IL-6 and TNF-α [5]. Therapeutic approaches directed at blocking some of these inflammatory mediators have proven effective in reducing patients’ symptoms.

Bai Xuan Xia Ta Re Pian (BXXTR) is a traditional Uighur medicine ancient prescription in China [6], which mainly consists of *Euphorbiae Humifusae Herba*, *Chebulae Fructus*, *Terminalia Belliricae Fructus*, *Chebulae Fructus Immaturus*, *Aloe*, *Resina Scammoniae*, and other components. BXXTR widely was used for the treatment of ringworm, tinea versicolor, psoriasis, atopic dermatitis, shingles, acne embolism etc. A large number of clinical practices showed that BXXTR presented favorable effect on the treatment of leukemia, including not only a short course, high curative rate and few side effects [7,8,9]. Some efforts have been made to combat psoriasis. Acitretin is a commonly used medicine for treatment of psoriasis and shows good curative effect. However, severe side effects of Acitretin limit its clinical use. For instance, Acitretin may cause elevation of serum liver enzymes, and triglyceride levels in some patients [10]. More than 90% of patients suffer from dry skin, itching, desquamation and dry mouth and other adverse reactions. Previous study has been proved that the combination of Acitretin with BXXTR could significantly reduce the dosage and adverse reactions [11]. Pharmacological studies confirmed that Acitretin could inhibit the differentiation and proliferation of Th17 cells both in vivo and vitro, and promote the conversion of Th0 cells into Treg cells, and regulate the balance between Th17 and Treg cells [12].

Imiquimod (IMQ) is a TLR7 and TLR8 immune activator for topical treatment of genital and perianal warts caused by actinic keratosis, superficial basal cell carcinoma and human papillomavirus [13]. Clinical data show that IMQ exacerbates the symptoms of already well-controlled psoriasis patients and can also induce symptoms at previously uninfected sites [14]. In recent years, it has been widely reported that IMQ was used as an effective tool drug to induce psoriasis-like skin inflammation mice model [15,16]. IMQ-induced skin lesions in mice are similar to those of psoriasis patients in terms of apparent and histological features and are collectively based on the IL-23/IL-17 Axis of the inflammatory pathways [17].

BXXTR is a traditional Uighur medicine ancient prescription in widely used in the treatment of psoriasis, presenting a high curative rate and few side effects. Given that the active constituents and action mechanism remain unclear, the aim of this study is to investigate the therapeutic efficacy of BXXTR in IMQ-induced psoriasis-like mice models and elucidate the potential active constituents and action mechanism of BXXTR. The results reveal that BXXTR could markedly alleviate IMQ-induced psoriasis by inhibiting IL-17-related inflammatory pathways. RNAseq analysis showed that BXXTR significantly induced the expression levels of 31 genes. Additionally, 75 compounds were determined by UPLC-TOF-MS analysis, and 11 compounds potentially targeted inflammatory related targets, which may be active constituents of BXXTR in antipsoriasis. These findings shed light on the understanding of the active constituents and action mechanism of BXXTR for the treatment of psoriasis and promoted the development of traditional Chinese medicine.

## 2. Results

### 2.1. BXXTR Treatment Alleviates IMQ-Induced Psoriasis-Like Skin Inflammation in BALB/c Mice

#### 2.1.1. PASI Scoring Analysis

**Figure 1:**
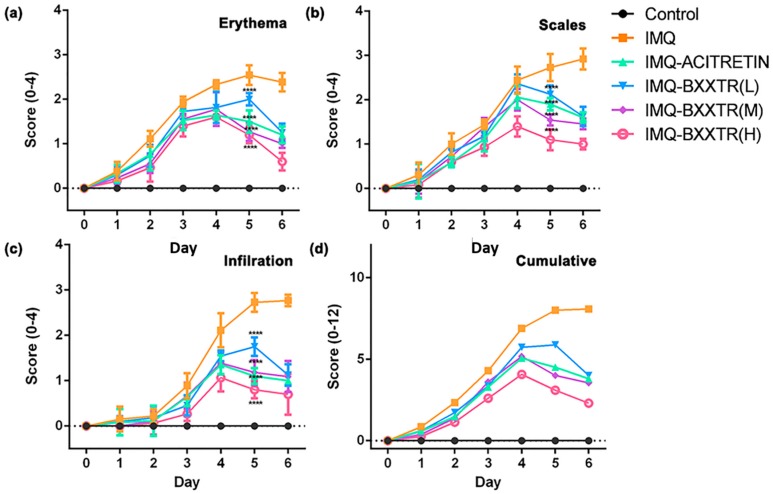
BXTTR treatment reduces IMQ-induced psoriatic-like cutaneous lesions in BALB/c mice on the back skin. (*n* = 10 for each group, black = control group, orange = IMQ group, cyan = IMQ-ACITRETIN group, blue = IMQ-BXXTR (L) group, purple = IMQ-BXXTR (M) group, pink = IMQ-BXXTR (H) group). Erythema, scales, infiltration of the back skin were scored on days 0–6 using the Psoriasis Area Severity Index (PASI) to assign a score of 0–4 to each animal and thereby assess the effects of daily treatment with IMQ cream and vehicle cream. (**a**) Erythema: data points are presented as group means ± SD; (**b**) scales: data points are presented as group means ± SD; (**c**) infiltration: data points are presented as group means ± SD; (**d**) cumulative score: erythema plus scales plus thickness. **** *p* < 0.0001 vs. IMQ.

To evaluate the antipsoriasis efficacy of BXXTR in vivo, we established an IMQ-induced psoriasis-like skin inflammation in BALB/c mice model. The severity of the psoriasis-like conditions in back skin of BALB/c mice was scored respectively on days 0–6 using the Psoriasis Area and Severity Index (PASI) clinical scoring system. Erythema and scales appeared first in IMQ-proceeded mice at the day 1 (Figure 1a,b); then infiltration (Figure 1c) was observed at day 2 in IMQ-treated group. During assay, the erythema, scales, and infiltration were most profound at days 5 and 6, and notably, erythema was decreased slightly at day 6 in the IMQ-treated group. Psoriasis-like skin inflammation was not observed on the back of control mice treated with Vaseline cream throughout the experimental period. Compared with the IMQ-treated group, IMQ-ACITRETIN and three IMQ-BXXTR groups exhibited considerable antipsoriasis effect with decreasing scores of erythema, scales, infiltration, and cumulative (Figure 1d). In addition, three IMQ-BXXTR groups showed a significant antipsoriasis effect in a dose-dependent way. In summary, the data reveal that BXTTR could significantly alleviate IMQ-induced psoriatic-like cutaneous lesions on the back skin of BALB/c mice.

#### 2.1.2. Back Skin and Ear Thickness

**Figure 2:**
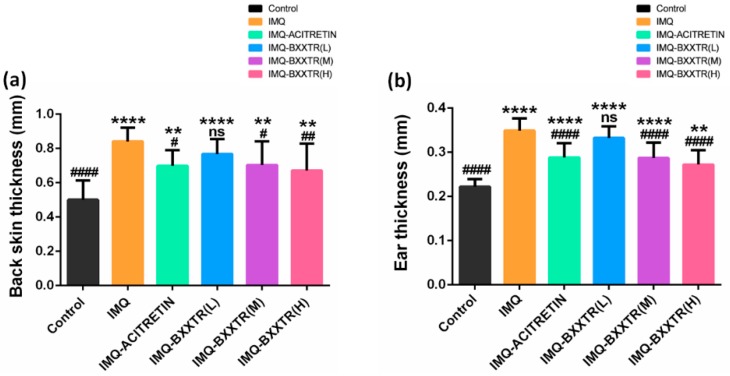
Survey thickness of back skin and right ear. The severity of psoriatic-like lesion induced by IMQ cream treatment was evaluated by measuring the thickness of the back skin and right ear. (**a**) Skin thickness on the backs of BALB/c mice; (**b**) right ear thickness of BALB/c mice. Bar diagram is expressed as group means ± SD of the skin or ear measurements on day 56 (*n* = 10 for each group). Column with * or ^#^ above manifests a significant difference between groups (*p* < 0.05) and “ns” means no significant difference. ** *p* < 0.01, **** *p* < 0.0001, vs. control; ^#^ *p* < 0.05, ^##^ *p* < 0.01, ^####^ *p* < 0.0001, vs. IMQ;

We used the thickness at day 5 of the back and ear as an indicator to assess the therapeutic effect of BXXTR. The back skin thickness of BALB/c mice was significantly increased by IMQ group compared with control group (0.8403 ± 0.08043 mm vs. 0.5003 ± 0.1114 mm; *p* < 0.0001). The back skin thickness of IMQ-ACITRETIN group was significantly reduced compared with IMQ group (0.6983 ± 0.09064 mm vs. 0.8403 ± 0.08043 mm; *p* = 0.0315). The back skin thickness in IMQ-BXXTR (M) group and IMQ-BXXTR (H) group was significantly lower than that of IMQ group. IMQ-BXXTR (M) vs. IMQ (0.7030 ± 0.1380 mm vs. 0.8403 ± 0.08043 mm; *p* = 0.0396); IMQ-BXXTR (H) vs. IMQ (0.6702 ± 0.1568 mm vs. 0.8403 ± 0.08043 mm; *p* = 0.0071). There was no significant difference between IMQ-BXXTR (L) group and IMQ group (Figure 2a). In addition, we measured the right ear thickness in BALB/c mice. Compared with control group, the ear thickness of IMQ group increased significantly (0.3491 ± 0.02737 mm vs. 0.2220 ± 0.0175 mm; *p* < 0.0001). In IMQ-ACITRETIN group, the thickness of the ear was significantly reduced compared with IMQ group (0.3100 ± 0.03559 mm vs. 0.3491 ± 0.02737 mm; *p* < 0.0001). The ear thickness of BALB/c mice in IMQ-BXXTR groups (M and H) was significantly reduced. IMQ-BXXTR (M) vs. IMQ (0.2873 ± 0.03495 mm vs. 0.3491 ± 0.02737 mm; *p* < 0.0001); IMQ-BXXTR (H) vs. IMQ (0.2720 ± 0.03293 mm vs. 0.3491 ± 0.02737 mm; *p* < 0.0001). There was no significant difference between IMQ-BXXTR (L) group and IMQ group (Figure 2b). The results suggested that BXXTR could abate psoriasis-like symptoms induced by IMQ in a dose-dependent manner.

### 2.2. Histological Examination

**Figure 3:**
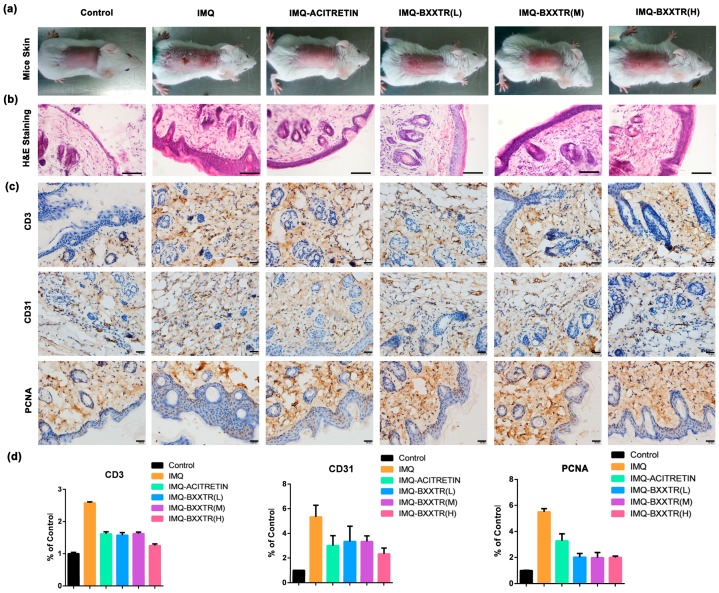
Presentation of the mice back skin phenotype, H&E staining and immunohistochemistry of sectioned skin. (**a**) Presentation of the phenotype of mice from the control group, IMQ group, IMQ-ACITRETIN group and IMQ-BXXTR group. The photograph is taken on day 6; (**b**) H&E-staining showed that IMQ-induced the back skin of mice showed dyskeratosis (nuclei in the stratum corneum) (Bar = 200 μm), thickening of the stratum spinosum layer, vasodilation, subcutaneous hemorrhage, inflammatory cells in the dermis infiltration compared with the control group; (**c**,**d**) immunohistochemistry of different groups. Compared with IMQ group, IMQ-ACITRETIN group and IMQ-BXXTR group alleviated IMQ-induced psoriasis-like symptoms from varying degrees (Bar = 20 μm).

To further evaluate the antipsoriatic-like symptoms effect, we displayed H&E staining and Immunohistochemistry of the different groups. Compared with control group, IMQ-induced psoriatic-like symptoms in the dorsal skin displayed dyskeratosis (nuclei in the stratum corneum), thickening of the stratum spinosum layer, vasodilation, subcutaneous hemorrhage, and inflammatory cells in the dermis infiltration. Compared with the IMQ-treated group, IMQ-ACITRETIN group and IMQ-BXXTR groups all exhibited obviously antipsoriasis-like lesions effect with the decrease of dyskeratosis (nuclei in the stratum corneum), thickening of the stratum spinosum layer, vasodilation, subcutaneous hemorrhage and inflammatory cells in the dermis infiltration (Figure 3a,b and Figure S2). Compared with control group, the IMQ-treated group displayed the higher degree of T lymphocyte infiltration (CD3), the higher proliferation rate of vascular endothelial cells (CD31) and the higher proliferation rate of epidermal keratinocytes (PCNA), which confirmed the psoriatic-like symptoms. Compared with the IMQ-treated group, the level of CD3, CD31 and PCNA of IMQ-ACITRETIN group and IMQ-BXXTR groups all decreased (Figure 3c,d). In summary, the results demonstrated BXXTR could inhibit IMQ-induced psoriatic-like symptoms.

### 2.3. RNAseq Analysis

**Figure 4:**
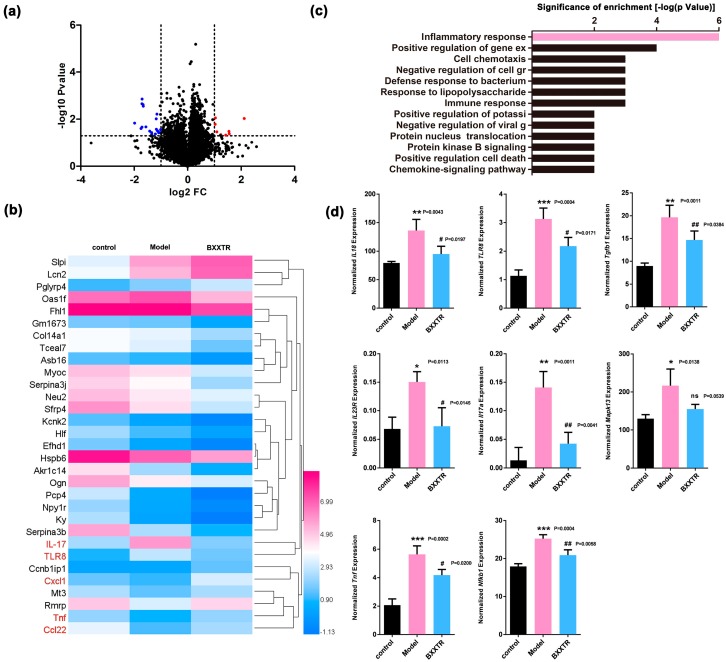
RNAseq analysis revealed that BXXTR could alleviate inflammatory reaction by inhibiting IL-17-related pathway. (**a**) Volcano plot showed the expression profiling changes by BXXTR treatment; (**b**) heat map showed the expression of genes that were up or down regulated by BXXTR treatment; (**c**) top signaling pathways affected by BXXTR-induced gene expression changes; (**d**) 8 genes were significantly changed and closely related to IL-17 related inflammation pathway. * *p* < 0.05, ** *p* < 0.01, *** *p* < 0.001, ^#^ *p* < 0.05, ^##^ *p* < 0.01.

The RNAseq analysis revealed that IMQ treatment induced a profound change in gene expression compared with control group with 237 probe sets having a *p* < 0.05 and fold change > 2 (83 upregulated and 154 downregulated) (Table S2). To determine the effect of BXXTR treatment, we compared IMQ-BXXTR group with IMQ group with 31 probe sets having a *p* < 0.05 and fold change > 2 (9 up-regulated and 22 down-regulated) (Figure 4a,b and Table S3). Among the 31 significantly altered genes, *IL-17A*, *TLR-8*, *TNF-α*, *CCl22* and *Cxcl1* were closely related to inflammation. We predicted IL-17-related inflammation pathway might be closely involved in BXXTR treatment (Figure 4c and Figure S1). Then we chose 78 probe sets with a fold change > 1.5 between IMQ group and IMQ-BXXTR group, we found 8 genes were significantly changed and closely related to IL-17-related inflammation pathway (Figure 4d). In summary, RNAseq analysis revealed BXXTR could alleviate inflammatory reaction by inhibiting IL-17-related pathway.

### 2.4. IL-17A and IL-23 Protein Levels in Serum and Skin Lesions by ELISA

**Figure 5:**
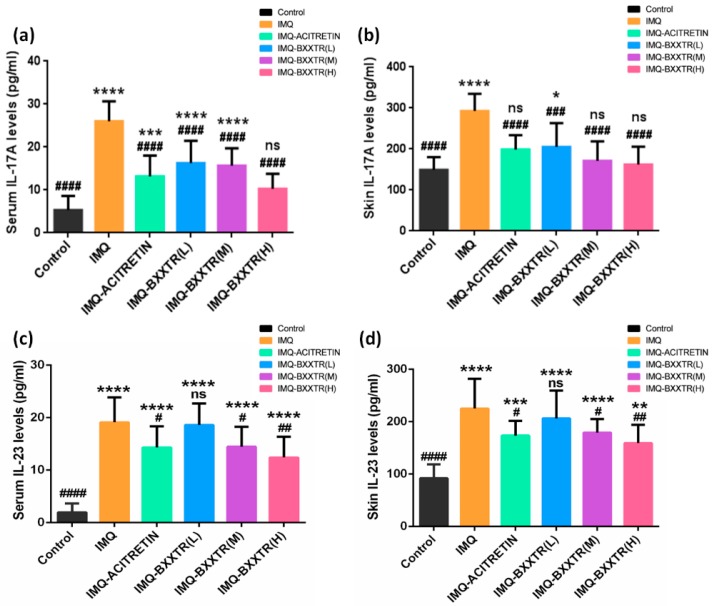
Effect of BXXTR on IMQ-induced changes in protein levels of IL-17A and IL-23 in the back skin and serum of BALB/c mice. (**a**) IL-17A protein levels in serum; (**b**) IL-17A protein levels in the back skin; (**c**) IL-23 protein levels in serum; (**d**) IL-23 protein levels in the back skin. IL-17A and IL-23 were measured using standard ELISA kits. Values are expressed as mean ± SD (*n* = 10 for each group). Column with * or ^#^ above manifests a significant difference between groups (*p* < 0.05) and “ns” means no significant difference. * *p* < 0.05, ** *p* < 0.01, *** *p* < 0.001, **** *p* < 0.0001, ^#^ *p* < 0.05, ^##^ *p* < 0.01, ^###^ *p* < 0.001, ^####^ *p* < 0.0001.

The level of IL-17A protein expression in serum of IMQ group was significantly higher than the control group (26.00 ± 4.637 pg/mL vs. 5.308 ± 3.323 pg/mL; *p* < 0.0001). IL-17A level was significantly decreased in the serum of IMQ-ACITRETIN group compared with IMQ group (13.18 ± 4.826 pg/mL vs. 26.00 ± 4.637 pg/mL; *p* < 0.0001). The expression levels of IL-17A in serum of three IMQ-BXXTR groups (L, M, H) were significantly lower than IMQ group in a dose-dependent manner, IMQ-BXXTR (L) vs. IMQ (16.26 ± 5.187 pg/mL vs. 26.00 ± 4.637 pg/mL; *p* < 0.0001); IMQ-BXXTR (M) vs. IMQ (15.64 ± 4.067 pg/mL vs. 26.00 ± 4.637 pg/mL; *p* < 0.0001); IMQ-BXXTR (H) vs. IMQ (10.29 ± 3.492 pg/mL vs. 26.00 ± 4.637 pg/mL; *p* < 0.0001). It is noteworthy that the level of IL-17A in the serum of IMQ-BXXTR (H) group was not significantly different from that in control group (Figure 5a). The expression level of IL-23 protein in serum of IMQ group was significantly higher than the control group (19.11 ± 4.742 pg/mL vs. 1.968 ±1.681 pg/mL; *p* < 0.0001). IL-23 levels were significantly decreased in the serum of IMQ-ACITRETIN group compared with IMQ group (14.93 ± 4.071 pg/mL vs. 19.11 ± 4.742 pg/mL; *p* = 0.0298). The expression level of IL-23 in serum of IMQ-BXXTR groups (M and H) was significantly lower than IMQ group in a dose-dependent manner, IMQ-BXXTR (M) vs. IMQ (14.46 ± 3.814 pg/mL vs. 19.11 ± 4.742 pg/mL; *p* = 0.0377); IMQ-BXXTR (H) vs. IMQ (12.42 ± 3.925 pg/mL vs. 19.11 ± 4.742 pg/mL; *p* = 0.0013). The expression level of IL-23 in the serum of IMQ-BXXTR (L) group was not significantly different from that in IMQ group (Figure 5c).

Similar changes can also be seen in the mice back skin IL-17A and IL-23 expression levels. The protein expression of IL-17A in the back skin of IMQ group was significantly higher than the control group (292.6 ± 42.39 pg/mL vs. 149.3 ± 30.88 pg/mL; *p* < 0.0001). IL-17A levels were significantly decreased in the skin of IMQ-ACITRETIN group compared to IMQ group (198.9 ± 34.71 pg/mL vs. 292.6 ± 42.39 pg/mL; *p* < 0.0001). Compared with IMQ group, the expression levels of IL-17A in skin of three IMQ-BXXTR groups (L, M, H) were significantly lower in a dose-dependent manner, IMQ-BXXTR (L) vs. IMQ (205.1 ± 5 8.02 pg/mL vs. 292.6 ± 42.39 pg/mL; *p* = 0.0002); IMQ-BXXTR (M) vs. IMQ (171.0 ± 47.86 pg/mL vs. 292.6 ± 42.39 pg/mL; *p* < 0.0001); IMQ-BXXTR (H) vs. IMQ (162.2 ± 43.54 pg/mL vs. 292.6 ± 42.39 pg/mL; *p* < 0.0001). It is worth noting that there was no significant difference in the protein expression of IL-17A in the skin of IMQ-ACITRETIN, IMQ-BXXTR (M) and IMQ-BXXTR (H) groups compared with control group (Figure 5b). The expression level of IL-23 in the back skin of IMQ group was significantly higher than the control group (225.09 ± 57.10 pg/mL vs. 92.22 ± 26.21 pg/mL; *p* < 0.0001). IL-23 levels were significantly decreased in the skin of IMQ-ACITRETIN group compared to IMQ group (173.5 ± 27.94 pg/mL vs. 225.09 ± 57.10 pg/mL; *p* = 0.0224); Compared with IMQ group, the expression of IL-23 in skin of IMQ-BXXTR groups (M and H) were significantly lower in a dose-dependent manner, IMQ-BXXTR (M) vs. IMQ (179.0 ± 26.00 pg/mL vs. 225.09 ± 57.10 pg/mL; *p* = 0.0495); IMQ-BXXTR (H) vs. IMQ (159.0 ± 35.10 pg/mL vs. 225.09 ± 57.10 pg/mL; *p* = 0.0022). The expression level of IL-23 in the skin of IMQ-BXXTR (L) group was not significantly different from that in IMQ group (Figure 5d).

### 2.5. BXXTR Could Decrease of IL-17A and IL-23 Levels in the Back Skin of BALB/c Mice

**Figure 6:**
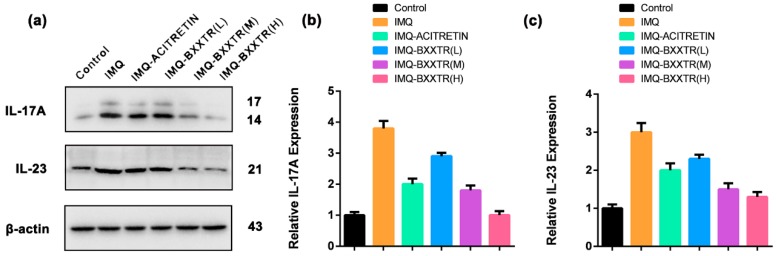
BXXTR could decrease of IL-17A and IL-23 levels in the back skin of BALB/c mice. (**a**–**c**) The expression of IL-17A and IL-23 in the back skin of BALB/c mice of different groups were measured with western blot.

To further verify the antipsoriasis-like symptoms of BXXTR, we checked the expression of IL-17A and IL-23 in the back skin of BALB/c mice. The expression levels of IL-17A and IL-23 of IMQ group were significantly upregulated than the control group. Compared with IMQ group, IL-17A levels were significantly decreased in IMQ-ACITRETIN group and three IMQ-BXXTR groups (L, M, H) (Figure 6a–c). Interestingly, higher does of BXXTR groups (M, H) exhibited better antipsoriasis-like symptoms with the relatively low level of IL-17A and IL-23.

### 2.6. The Active Constituent Analysis and Prediction of BXXTR

To characterize the constituents of BXXTR, we collected a large number of chemical components that had been identified and analyzed (*Euphorbiae Humifusae Herba*, *Chebulae Fructus*, *Terminalia Belliricae Fructus*, *Chebulae Fructus Immaturus*, *Aloe,* and *Resina Scammoniae*) and established the chemical composition library of BXXTR. Using UPLC-TOF-MS, 75 chemical components were detected in BXXTR by comparing the exact molecular mass and their chromatographic behaviors, including flavonoids, tannins, triterpenoids, phenols, anthraquinones and their derivatives, chromones, naphthalenes, pyrones and so on (Figures S3–S9 and Table S1).

To further classify the active constituents of BXXTR as anti-inflammatory, the potential targets of 75 compounds determined by UPLC-TOF-MS were predicted by the SEA SEARCH SERVER [18]. Next, the functional clustering of these potential targets was conducted through DAVID web server [19]. The results revealed that 11 of 75 compounds’ potential targets were closely associated with inflammation, which was mostly consistent with the results of RNAseq (Table 1). These findings indicated that the 11 compounds might be the potential active constituents of BXXTR in anti-inflammatory.

## 3. Discussion

According to incomplete statistics, there are 202 kinds of national medicines that have been included in the national pharmacopeia, of which 115 are medicinal herbs and 87 are compound preparations. Due to factors such as complex chemical components or unclear pharmacological mechanisms, their development has been constrained. The research ideas of “multicomponent, multitarget and cotreatment” were used in this experiment to screen specific targets of the compound preparations and related intervention pathways to explore the mechanism of BXXTR in the treatment of psoriasis [20,21,22].

Psoriasis is characterized by thickening of the prickle cell layer, parakeratosis, and massive inflammatory cell infiltration. Symptoms reflected on the skin are a large number of scales and raised erythema. Increased expression of IL-23 and Th17-associated cytokines in serum and lesion sites of patients with psoriasis, suggesting that IL-23/IL-17 inflammation axis plays a key role in psoriasis [23].

In recent years, a mouse model of psoriasis lesions induced by IMQ has been widely used. IMQ as a ligand agonist of TLR7 and TLR8 induces similar psoriatic skin lesions in mice [15]. Thomas Nordstrøm Kjær et al. compared the RNA microarray data of IMQ-induced models of psoriatic skin lesions of mice with psoriasis patients, confirming that their gene expression changes are comparable [24].

The PASI scoring system was used to evaluate the severity of skin lesions in terms of erythema, scales and infiltration. The actual thickness of the back skin and right ear of mice was measured using a vernier caliper. The results showed that IMQ-ACITRETIN group and IMQ-BXXTR group could significantly improve the severity of psoriatic lesions compared with the model group, and the treatment effect presented a dose-dependent trend in IMQ-BXXTR group. The results of pathological sections showed that IMQ group exhibited dyskeratosis, subcutaneous hemorrhage, and inflammatory cell infiltration compared with control group. BXXTR can inhibit the severity of these symptoms in different degrees.

Emerging studies have demonstrated that IL-23 secreted by DCs and macrophages in the lesions of psoriasis patients can induce Th17 lymphocyte activation and release pro-inflammatory cytokine IL-17. IL-17 synergizes with Interferon-γ (IFN-γ) and TNF-α to stimulate keratinocyte proliferation and secrete a large number of inflammatory cytokines such as IL-6, TGF-β, IL-18, IL-17, IL-12, IL -15, TNF-α, GM-CSF, ICAM-1, etc. These inflammatory cytokines can actively recruit and activate neutrophils, accelerate the accumulation of T cells into the epidermis, leading to epidermal hyperplasia, acanthosis and hyperkeratosis [17,25,26]. Some inflammatory cytokines such as IL-6, TGF-β, IL-23 can continuously promote the differentiation of Th17 lymphocytes [27] and amplify the cascade response. IL-17 binds to the receptor and is further involved in the inflammatory response through the MAPK and the NF-κB signaling pathway [28,29,30,31].

**Figure 7:**
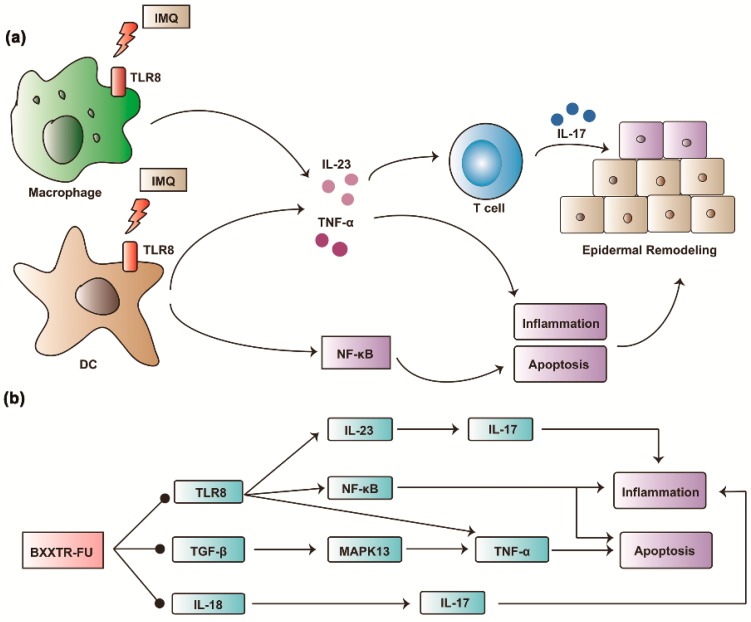
Schematic diagram of the anti-inflammation mechanism of BXXTR in IMQ-induced psoriasis-like skin inflammation. (**a**) IMQ stimulate TLR8 in macrophage and DCs thus to release IL23, TNF-α and NF-κB to induce IL-17 dependent inflammation; (**b**) BXXTR could inhibit the expression of TLR8, TGF-β and IL-18 thus to inhibit the activation of TLR8-IL-23-IL-17, TLR8-NF-κB, TGF-β-MAPK13-TNF-α and IL-18-IL-17 signaling pathways to inhibit apoptosis and inflammation.

In present study, RNAseq and KEGG database analysis of skin lesions of the control group, IMQ group and IMQ-BXXTR group was performed. IMQ stimulated TLR8 in macrophage and DCs thus to release IL-23, TNF-α and NF-κB to induce IL-17 dependent inflammation (Figure 7a). BXXTR could inhibit the expression of TLR8, TGF-β and IL-18, inhibiting the activation of TLR8/IL-23/IL-17, TLR8/ NF-κB, TGF-β/MAPK13/TNF-α and IL-18/IL-17 signaling pathways to inhibit apoptosis and inflammation (Figure 7b). Given the fact that BXXTR could inhibit IMQ-Induced psoriasis-like skin inflammation, BXXTR might represent a new avenue for therapeutic intervention.

An ELISA kit was used to detect the expression levels of IL-17A and IL-23 protein in mice skin lesions and serum. Compared with the IMQ group, the expression level of IL-17A and IL-23 protein in IMQ-BXXTR group and IMQ-ACITRETIN group were significantly reduced. Interestingly, there was no significant difference in IL-17A protein expression levels in serum between IMQ-BXXTR (H) group and control group. The expression of IL-17A protein in the lesions in the IMQ-ACITRETIN group; the IMQ-BXXTR (M) group and IMQ-BXXTR (H) group was not significantly different from that in the control group. It was demonstrated that BXXTR significantly reduced the expression of IL-17A and IL-23 protein in mice skin lesions and serum.

In conclusion, studies have shown that IMQ induces psoriasis-like skin inflammation through the IL-23/IL-17 inflammatory axis. The PASI scoring, measurement of back skin and ear thickness, and histological analyses showed that BXXTR dose-dependently alleviated IMQ-induced psoriasis-like skin lesions. Based on RNAseq technology and KEGG database analysis, BXXTR significantly reduced the severity of skin lesions by inhibiting IL-17-related inflammatory pathways. The ELISA and western blot data further validated the conclusions in protein level expression. In addition, we identified 75 compounds from BXXTR by UPLC-TOF-MS, and the potential targets of these compounds were predicted by the SEA SEARCH SERVER. The functional clustering of these potential targets was conducted through DAVID web server. The results revealed that 11 of 75 compounds’ potential targets were closely associated with inflammation, which was mostly consistent with the results of RNAseq. These findings indicated that the 11 compounds might be the potential active constituents of BXXTR in anti-inflammatory. These data indicate that BXXTR could combat psoriasis inflammation by multiple targets and multiple pathways synergistically. This study could provide important guidance for the elucidation of the active constituents and action mechanism of BXXTR for the treatment of psoriasis.

## 4. Materials and Methods

### 4.1. Preparation of Extract from BXXTR

*Euphorbia Humifusae Herba*, *Chebulae Fructus*, *Terminalia Belliricae Fructus*, *Chebulae Fructus Immaturus*, *Aloe* and *Resina Scammoniae* were purchased in Urumqi. The extract of BXXTR was prepared by strictly following the prescription preparation process [6].

### 4.2. Psoriasis-Like Skin Model

Animal welfare and the experimental procedures were in accordance with the Ethical Regulations on the Care and Use of Laboratory Animals of Shihezi University, and all animal experiments were performed with the approval and under the guidelines of the Animal Experimental Ethics Committee of the First Affiliated Hospital of medical college, Shihezi University (A2012-016, 30 May 2012). Specific pathogen-free male BALB/c mice, aged 6–8 weeks old, were purchased from Changsheng Biotechnology Co., Ltd. (Certificate No. SCXK (Liao) 2010–0001, Benxi, China). Before the start of any experimental procedures, the animals were shaved back and given 2 days of acclimatization. Throughout the experiment, animals had access to an unlimited amount of water and feed.

The 60 BALB/c mice were randomly divided into 6 groups: control group (*n* = 10), IMQ-treated group (*n* = 10), IMQ-ACITRETIN-treated group (*n* = 10), IMQ-BXXTR(L) group (*n* = 10), IMQ-BXXTR(M) group (*n* = 10), IMQ-BXXTR(H) group (*n* = 10). Based on previous reports [32], IMQ-treated and three treatment groups of mice received a daily dose of 62.5 mg of a 5% IMQ cream (Aldara; MEDA AS) topical applied evenly to their back and outside of the right ear for 0–6 days. The control group received a similar daily dose of vehicle cream (Vaseline Lanette cream; Fagron, Rotterdam, The Netherlands).

IMQ-BXXTR(L), IMQ-BXXTR(M) and IMQ-BXXTR(H) groups were administered at 33.0, 68.0, 134.0 mg/kg/day of BXXTR for 0–6 days by oral gavage respectively. For IMQ-ACITRETIN-treated group, Acitretin (Acitretin Capsules purchased from Huapont Pharm, Chongqing, China) was used as a positive control drug at 84.0 mg/kg, twice a day in interval 8 h manners (namely total 168 mg/kg/day) by oral gavage for 0–6 days. On the day 6, the animals were euthanized and the shaved the back skin were immediately resected. The tissues were fixed in 2 times volumes of 4% paraformaldehyde for 24 h and embedded in paraffin for histological analysis. The remaining lesion tissue was quickly frozen in liquid nitrogen and stored at −80 °C.

### 4.3. Scoring Severity of Skin Lesion

Based on the clinical Psoriasis Area and Severity Index (PASI), all animals were assessed for the severity of the psoriasis-like skin condition on days 0–6. Erythema, scales, infiltration were scored independently from 0 to 4 as follows: 0, none; 1, slight; 2, moderate; 3, marked; 4, very marked. The cumulative score is the sum of the three elements, indicating the severity of inflammation (scale 0–12).

### 4.4. Measurement of Back Skin and Ear Thickness

The back skin and ear thickness were measured using a vernier caliper (MNT-150, Shanghai, China). Three replicates were performed, the data of the back and ear was used to evaluate epidermal proliferation and inflammation.

### 4.5. Histological Analyses

The paraffin-embedded tissues in different groups were sectioned and hematoxylin and eosin (H&E) stained for histological evaluation. The samples were fixed in 10% formalin and embedded in paraffin. The paraffin-embedded specimens were sectioned into 5-μm thick slices, deparaffinized in xylene for 15 min, hydrated in a graded alcohol series, and rinsed three times with 1% PBS. The slices were stained with hematoxylin and eosin (H&E) for microscopic observations according to the manufacturer’s standard protocols. The sections were examined under a Nikon 80i plus confocal laser-scanning microscope (Nikon, Tokyo, Japan) at ×100 magnification.

### 4.6. RNA Isolation, Library Preparation for RNAseq

Total RNA was isolated from the epidermal and dermal fractions of the skin, using RNeasy Fibrous Tissue Mini-Kit (Qiagen, Germantown, MD, USA). The RNA concentration was determined by NanoDrop, the integrity of the RNA was checked by agarose gel electrophoresis, and the RIN value of the RNA was determined by an Agilent 2100 Bioanalyzer. After the quality control of the RNA sample was completed, oligo-dT magnetic beads were used to separate mRNA from the total RNA and perform Purification. After fragmentation of the mRNA, 15 cycles of PCR amplification were performed using universal primers complementary to the adapter sequence to complete construction of the RNA library.

The RNA library under test completes cluster generation on the cBot and the flowcell is then transferred to the HiSeq 2000 sequencing system for next-generation sequencing and data analysis following the Illumina standard protocol, RNAseq data were aligned using STAR, the reference genome was Mus musculus genome version 10 (GRCm38), and the expression was quantified using Cufflinks 2.2.1, and the difference was analyzed using Cuffdiff. R/Bioconductor packages including DESeq2 were used for gene expression analysis [33]. The volcano plot was drawed by GraphPad and the heatmap was gained by R heatmaply package.

### 4.7. Measurement of IL-17A and IL-23 in Skin and Serum by ELISA

On day 7, the tissue of BALB/c mice back skin and serum were collected, snap frozen in liquid nitrogen and stored at −80 °C. Tissues were collected from each treatment and suspended in RIPA lysis buffer. The lysed tissue was centrifuged at 12,000 rpm for 10 min, the supernatant was collected, and protein content was determined using a BCA Protein Assay Kit (Beyotime, Shanghai, China). Mouse IL-17A Precoated ELISA kit (Dakewe Bio-engineering Co., Ltd., Shenzhen, China) was used to quantitatively detect the protein expression levels of IL-17A and IL-23 in the sample. Measurement wavelength is 450 nm.

### 4.8. Immunohistochemistry Analysis (IHC)

Sections of the mice skin were submerged in EDTA antigenic retrieval buffer (pH 8.0) or citrate buffer (pH 6.0), and microwaved for antigenic retrieval. The slides were then incubated with rabbit anti-CD3, rabbit anti-CD31, and rabbit anti-PCNA polyclonal antibody (1:400) for 30–40 min at 37 °C respectively. Normal rabbit/mouse IgG was used as a negative control. The slides were then treated by HRP polymer conjugated secondary antibody for 30 min and developed with diamino-benzidine solution. Meyer’s hematoxylin was used as a counterstain.

### 4.9. Western Blot

The tissue of BALB/c mice back skin were collected, snap frozen in liquid nitrogen and stored at −80 °C. Tissues were collected from each treatment and suspended in RIPA lysis buffer. The lysed tissue was centrifuged at 12,000 rpm for 10 min, the supernatant was collected, and protein content was determined using a BCA Protein Assay Kit (Bio-Rad Laboratories, Hercules, CA, USA). Equal amounts of the total protein were separated by 10–15% SDS-PAGE and transferred to PVDF membranes, the membranes were soaked in blocking buffer (5% skimmed milk or BSA). Proteins were detected using primary antibodies, followed by HRP-conjugated secondary antibody and visualized by using ECL as the HRP substrate. Quantification of immunoblot was performed by Quantity One 4.4.

### 4.10. UPLC-TOF-MS Analysis

UPLC-TOF-MS: Waters ACQUITY UPLCTM system connected to Waters LCT Premier XE time-of-flight mass spectrometer (Waters, New York, NY, USA). Chromatography was performed using an ACQUITY UPLC^®^ BEH C18 column (2.1 × 50 mm, 1.7 μm), and the column temperature was maintained at 30 °C. The mobile phase consists of solvent A (methanol) and B (0.1% formic acid aqueous) by gradient elution. The gradient program is as follows: 0.7–4.1 min, 10–30% A; 4.1–10.7 min, 30–45% A; 10.7–15.7 min, 45–75% A; 15.7–16.1 min, 75–95% A. The flow rate was 0.2 mL/min. The auto-sampler pool was conditioned at 4 °C. The injection volume is 2 µL. UPLC-TOF-MS was used to gather the chromatogram of samples in ES^+^/ES^−^ ions mode. MassLynx V4.1 chromatographic workstation was used to process data.

### 4.11. The Prediction of Active Constituents of BXXTR

The potential targets of all the compounds from BXXTR determined by UPLC-TOF-MS were predicted by utilizing the SEA SEARCH SERVER. The predicted targets were clustered by gene ontology to obtain special targets.

### 4.12. Statistical Analysis

The data are expressed as means ± SD. and analyzed with GraphPad Prism 6.0. Statistical comparisons were made by one-way ANOVA. *p* < 0.05 was considered statistically significant.

## Figures and Tables

**Table 1:** Potential anti-inflammatory constituents by target prediction.

No.	Source	Compound	Structure	R.t (min)	Quasi-Molecular Ion	Measurements	Error (ppm)	Predicted Targets
5	*Euphorbiae Humifusae Herba*	3,3′-2-di-*O*-methyl ellagic acid-4-*O*-β-d-glucopyranoside	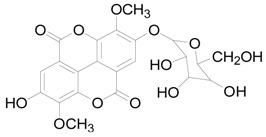	6.81	[M − H]^−^	491.0825	−0.2	ADORA2A, ADORA1, IL1F8, S100A8, HCK
10	*Euphorbiae Humifusae Herba*	7′-ethyl-sanguisorbic acid dilactone	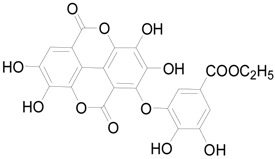	9.63	[M − H]^−^	497.0313	1.8	ADORA2A, ADORA1, SERPINE1, HSPA1A, TNF
11	*Euphorbiae Humifusae Herba*	1-(2′3′4′5′-tetrahydroxypentyl)-6,7-dimethyl-quinoxaline-2,3-(1H,4H)-dione	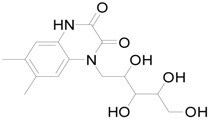	4.42	[M − H]^−^	323.1242	−0.3	ADORA2A, ADORA1, CCL19, FPR2, CCL5, CCL4
19	*Terminaliae Belliricae Fructus*	3,3′-di-*O*-methylellagic acid	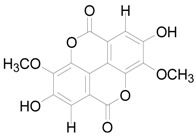	11.42	[M − H]^−^	329.0298	0.3	GSK3A, GSK3B, LYN, CELA1, SYK, ESR1
25	*Chebulae Fructus and Chebulae Fructus Immaturus*	chebuloside II	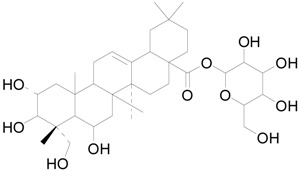	11.47	[M + COOH]^−^	711.3954	−0.3	ESR1, STAT1
36	*Aloe*	aloesaponol I-6-*O*-β-d-glucoside	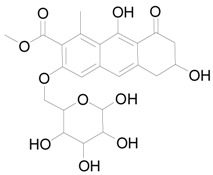	13.45	[M + COOH]^−^	523.1411	−3.4	ADORA2A, ADORA1, SYK, ESR1
55	*Aloe*	plicataloside	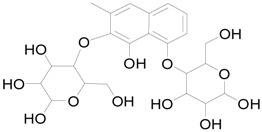	9.79	[M + COOH]^−^	559.1699	2.5	ADORA2A, ADORA1, CCL22
56	*Aloe*	aloveroside A	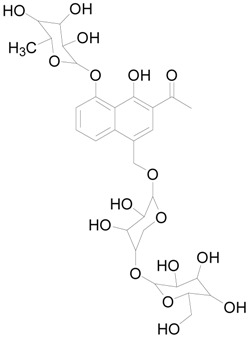	7.09	[M − H]^−^	671.2172	−2.2	ADORA2A, ADORA1, LYN, CELA1,
57	*Aloe*	aloveroside B	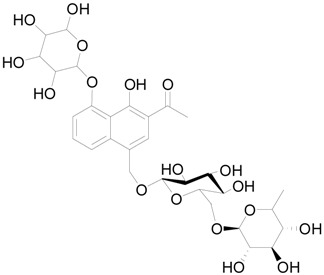	6.45	[M + COOH]^−^	733.2145	−4.0	ADORA2A, ADORA1, IL1F6, IL1RL1, TNF
58	*Aloe*	8-(α-l-rhamnopyranosyloxy)-3-(β-d-xylopyranosyl oxymethyl)naphthalen-ol	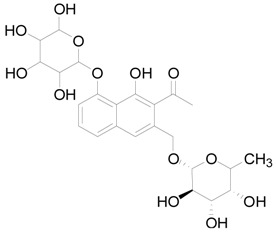	7.77	[M + COOH]^−^	571.1642	−3.7	PTPN2, ADORA2A, ADORA1
74	*Aloe*	feroxin B	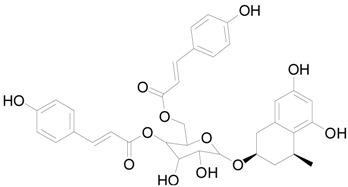	12.13	[M + Na]^+^	671.2049	−4.7	PRKCA, SRC, CXCL1

## Supplementary Materials

Supplementary materials can be found at http://www.mdpi.com/1422-0067/19/7/1822/s1.
