# Supplementary material for: Decryption of Active Constituents and Action Mechanism of the Traditional Uighur Prescription (BXXTR) Alleviating IMQ-Induced Psoriasis-Like Skin Inflammation in BALB/c Mice
Source: Int J Mol Sci. 2018 Jun 21;19(7):1822. doi: 10.3390/ijms19071822 (PMC6073889; doi:10.3390/ijms19071822)
Supplement: Supplementary file 1 [file ijms-19-01822-s001.zip › Support information 20180610.pdf]

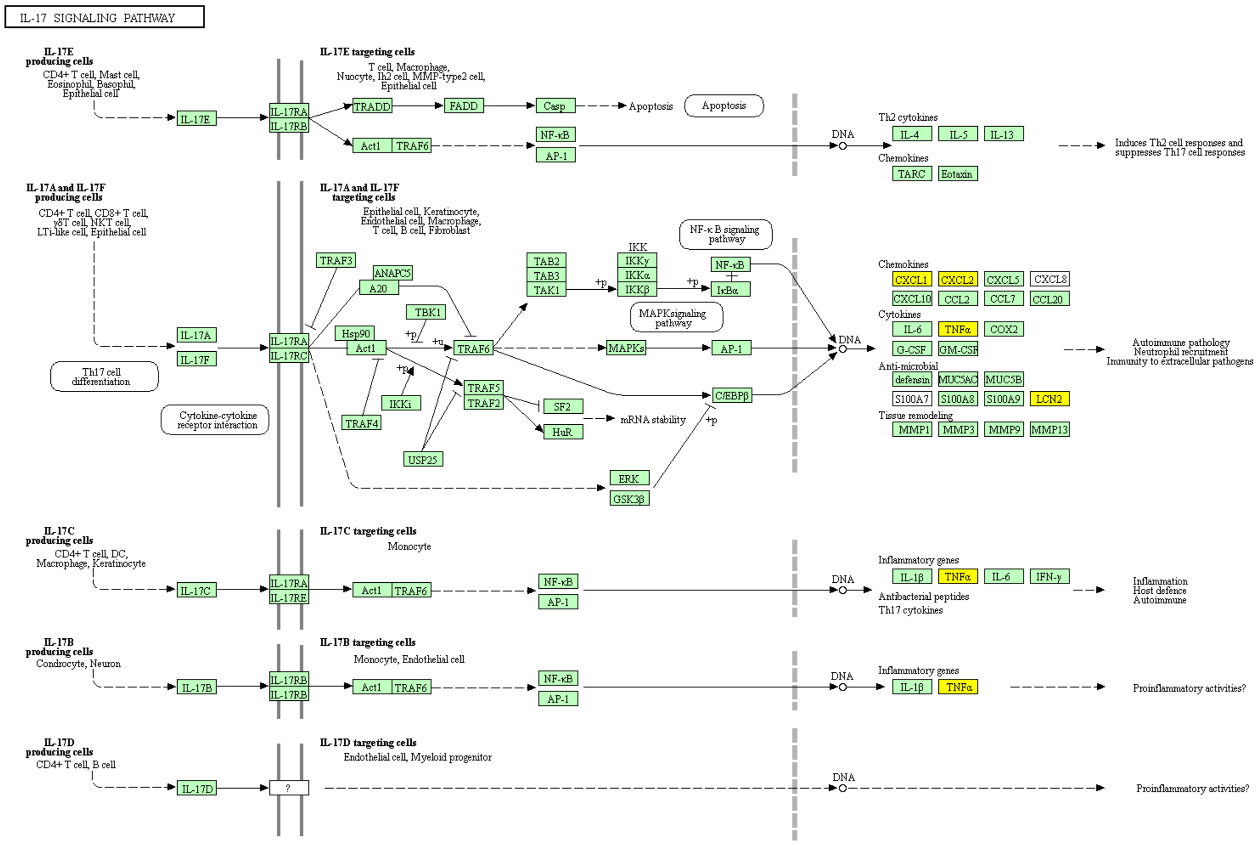

Figure S1 The analysis of BXXTR-affected pathway by KEGG.

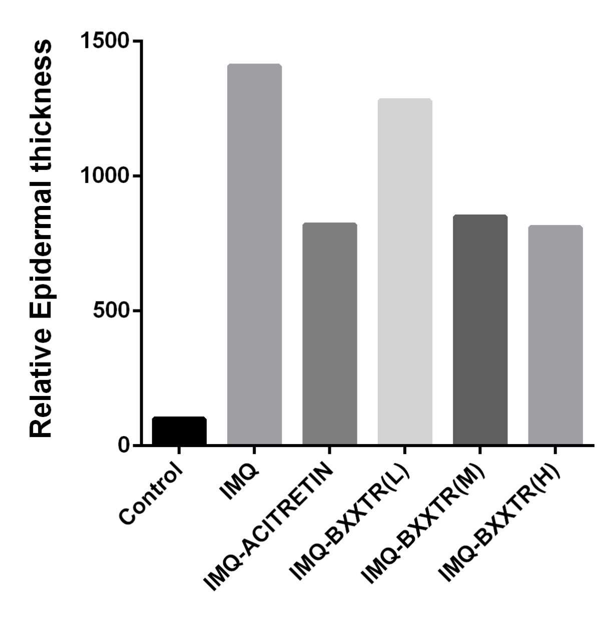

Figure S2 The analysis of the epidermal thickness.

Table S1 Analysis of the chemical constituents of BXXTR by UPLC-TOF-MS

N.O	Source	Compound	Structure	M.W
1	*Euphorbiae Humifusae Herba*	Quercetin	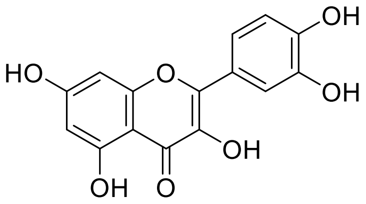	302.0427
2	*Euphorbiae Humifusae Herba*	Brevifolin	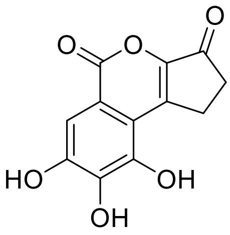	248.0321
3	*Euphorbiae Humifusae Herba*	Ellagic acid	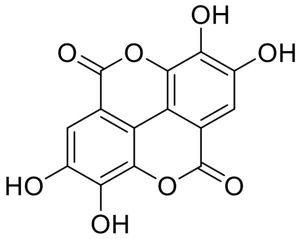	302.0063
4	*Euphorbiae Humifusae Herba*	Ethyl brevifolin carboxylate	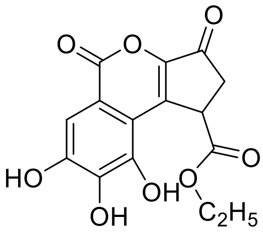	320.0532
5	*Euphorbiae Humifusae Herba*	3, 3′-2-di-O-methyl ellagic acid-4-O-β-D-glucopyranoside	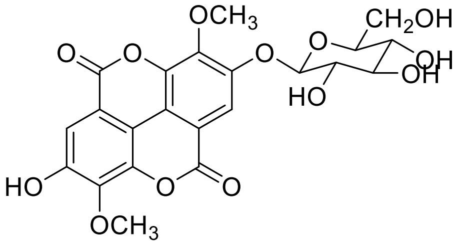	492.0904
6	*Euphorbiae Humifusae Herba*	Ethyl gallate	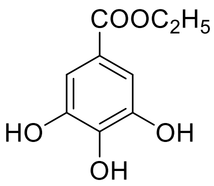	198.0528
7	*Euphorbiae Humifusae Herba*	Brevifolin carboxylic acid	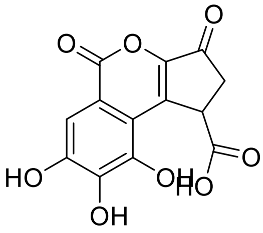	292.0219

8	*Euphorbiae Humifusae Herba*	Gallic acid	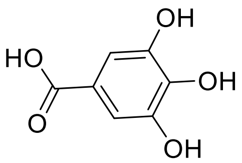	170.0215
9	*Euphorbiae Humifusae Herba*	Methyl gallate	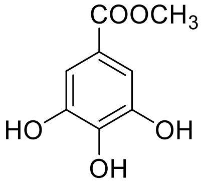	184.0372
10	*Euphorbiae Humifusae Herba*	7''-ethyl-sanguisorbic acid dilactone	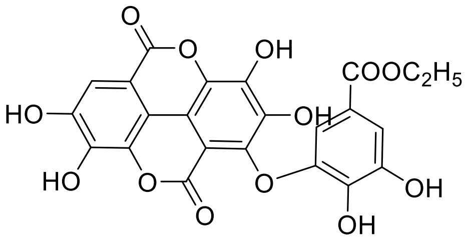	498.0434
11	*Euphorbiae Humifusae Herba*	1-(2'3'4'5'-tetrahydroxypentyl)-6,7-dimethyl-quinoxaline-2,3-(1H,4H)-dione	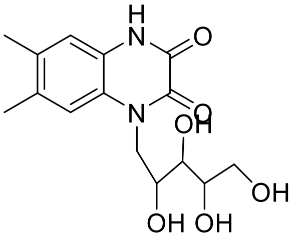	324.1321
12	*Euphorbiae Humifusae Herba*	Euphormisin M2	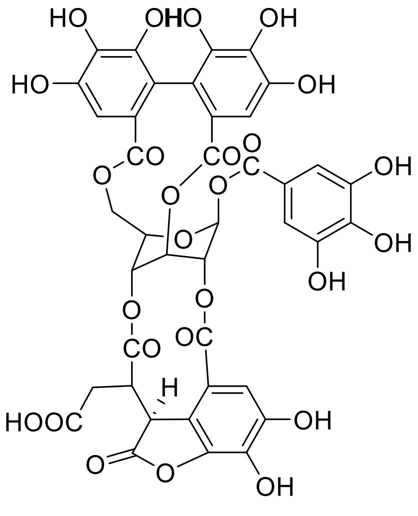	924.0869
13	*Euphorbiae Humifusae Herba*	Luteolin-7-O-β-D-glucopyranoside	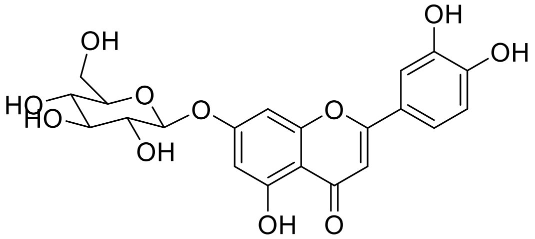	448.1000

14	*Euphorbiae Humifusae Herba*	Methyl brevifolin carboxylate	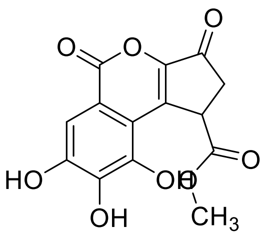	306.0376
15	*Euphorbiae Humifusae Herba*	Apigenin-7-O-β-D-lutinoside	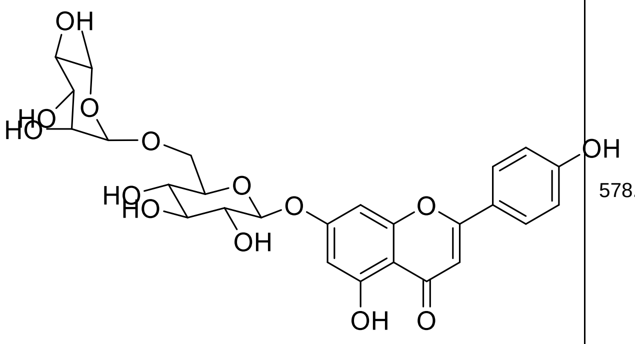	578.1636
16	*Euphorbiae Humifusae Herba*	Apigenin-7-O-β-D-apiofuranosyl-(1-2)-β-D-glucopyranoside	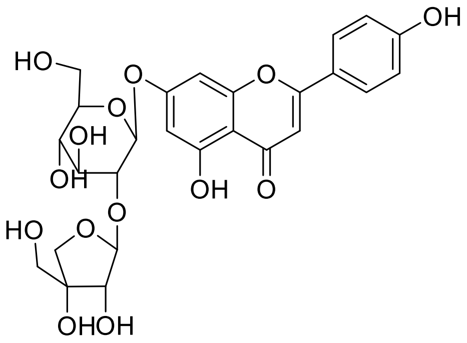	564.1479
17	*Euphorbiae Humifusae Herba*	Furosin	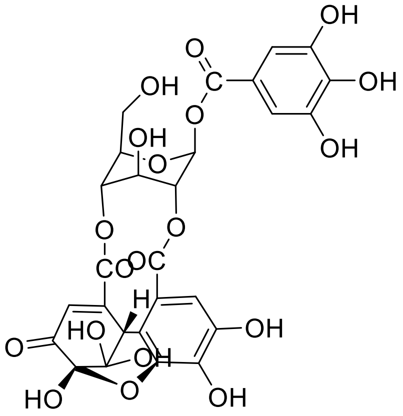	650.0755
18	*Euphorbiae Humifusae Herba*	3-oxo-7,8-dihydro-a-ionone-11-O-b-glucoside	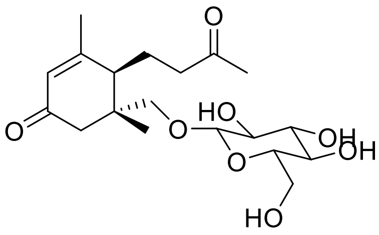	386.1941

19	*Terminaliae Belliricae Fructus*	3,3'-di-O-methylellagic acid	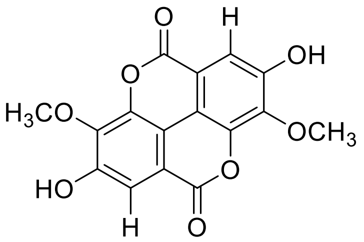	330.0376
20	*Chebulae Fructus, Terminaliae Belliricae Fructus* and *Chebulae Fructus Immaturus*	3,4,8,9,10-pentahydroxydibenzo-[β,D]pyran-6-one	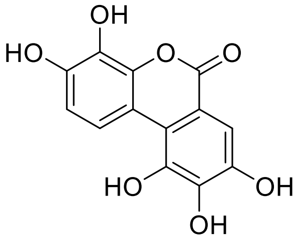	276.027
21	*Terminaliae Belliricae Fructus* and *Chebulae Fructus Immaturus*	(S)-Flavogallonic acid	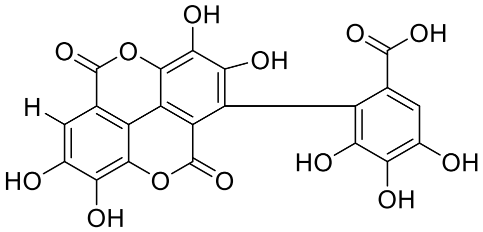	470.0121
22	*Terminaliae Belliricae Fructus*	3,3'-di-O-methyl-4-O-(β-D-xylopyranosyl)ellagic acid	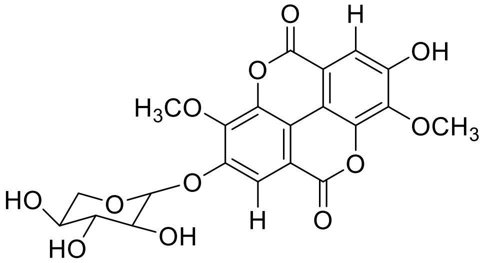	462.0798
23	*Chebulae Fructus* and *Chebulae Fructus Immaturus*	Arjungenin	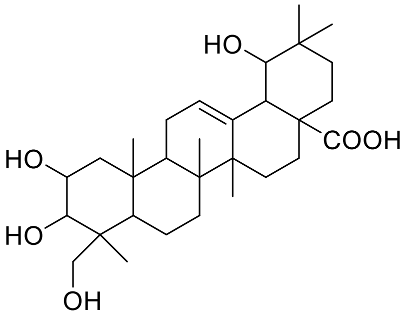	504.3451
24	*Chebulae Fructus, Terminaliae Belliricae Fructus* and *Chebulae Fructus Immaturus*	Terflavin D	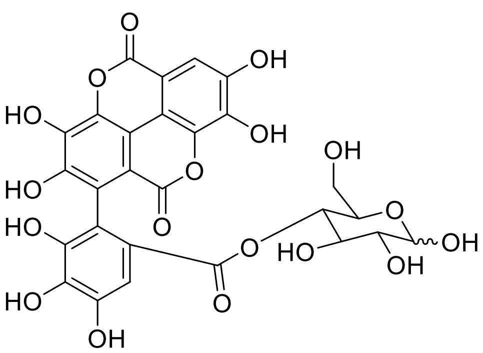	632.065

25	*Chebulae Fructus* and *Chebulae Fructus Immaturus*	Chebuloside Ⅱ	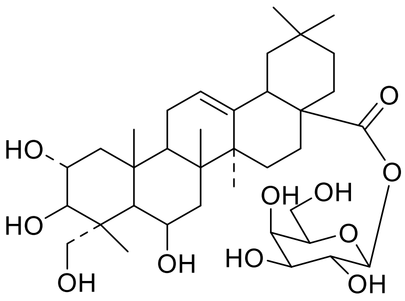	666.3979
26	*Chebulae Fructus*, *Terminaliae Belliricae Fructus* and *Chebulae Fructus Immaturus*	Chebulagic acid	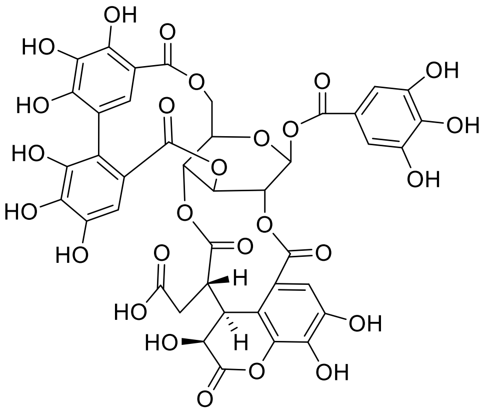	954.0975
27	*Chebulae Fructus*, *Terminaliae Belliricae Fructus* and *Chebulae Fructus Immaturus*	1,3,6-tri-O-galloyl-β-D-glucose	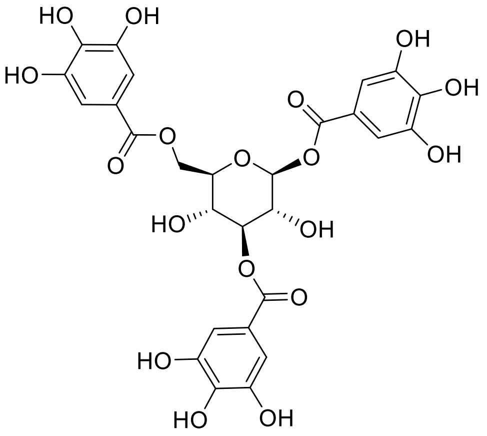	636.0963
28	*Chebulae Fructus*, *Terminaliae Belliricae Fructus* and *Chebulae Fructus Immaturus*	1,2,3,4,6-penta-O-galloyl-β-D-glucose	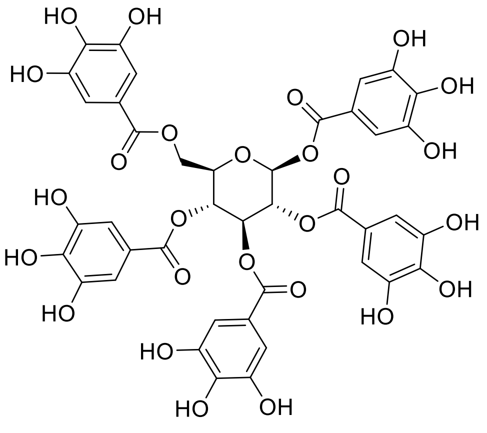	940.1182

29	*Chebulae Fructus, Terminaliae Belliricae Fructus* and *Chebulae Fructus Immaturus*	Corilagin	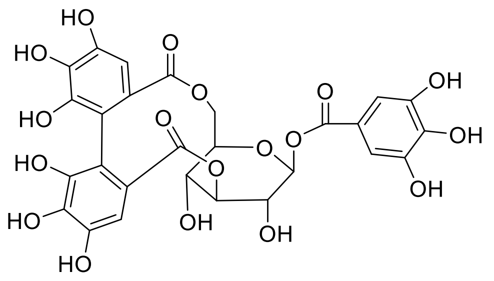	634.0806
30	*Chebulae Fructus* and *Chebulae Fructus Immaturus*	Arjunolic acid	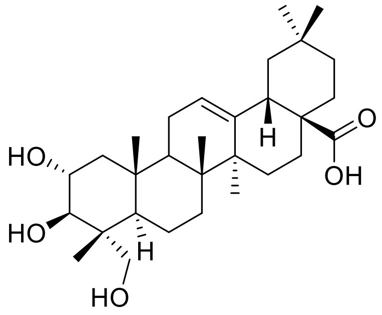	488.3502
31	*Chebulae Fructus, Terminaliae Belliricae Fructus* and *Chebulae Fructus Immaturus*	Triethyl chebulate	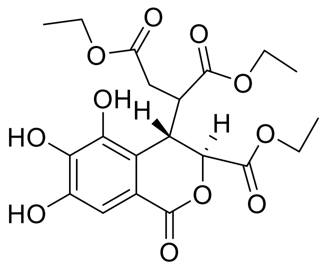	440.1319
32	*Aloe*	Rutin	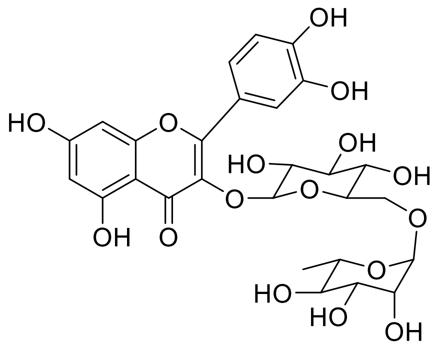	610.1534
33	*Aloe*	Aloe-emodin diglucoside	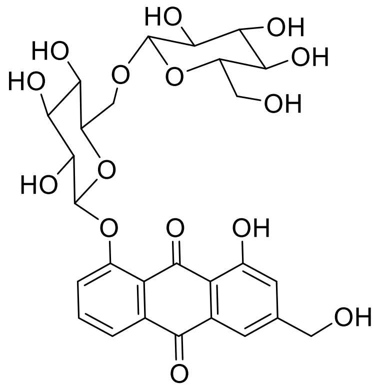	594.1585

34	*Aloe*	Chrysophanol glucoside	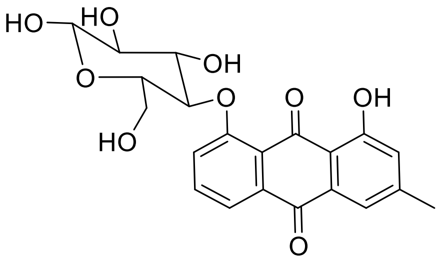	416.1107
35	*Aloe*	Aloenoside	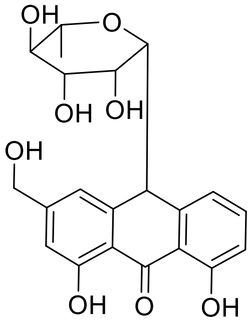	402.1315
36	*Aloe*	Aloesaponol-6-O-β-D-glucoside	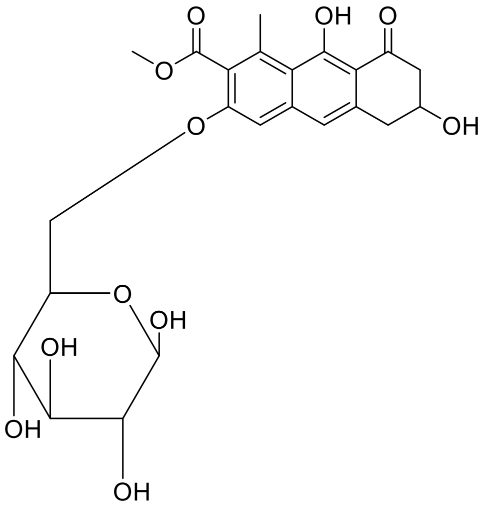	478.1475
37	*Aloe*	Littoraloin	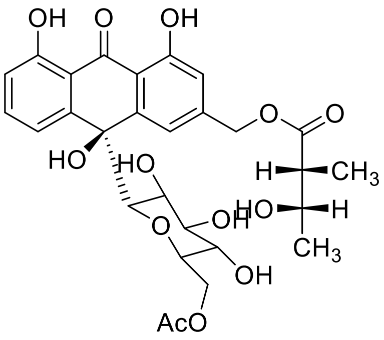	576.1843

38	*Aloe*	Aloenin B	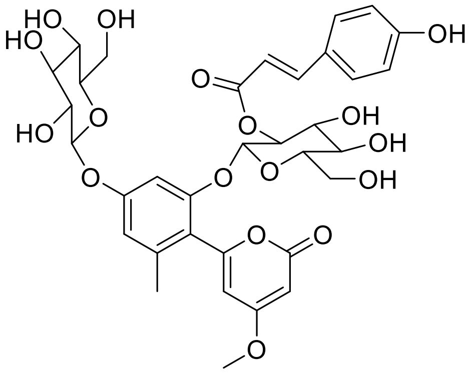	718.2109
39	*Aloe*	10-O-β-D-glucopyranosyl aloenin	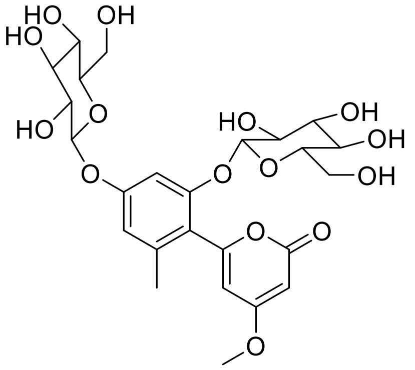	572.1741
40	*Aloe*	8-C-glucosyl-(S)-aloesol	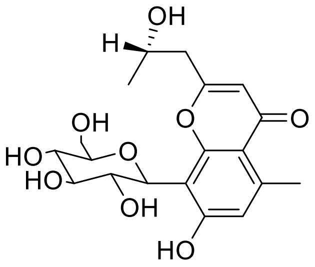	396.142
41	*Aloe*	8-C-glucosyl-7-O-methyl-(S)-aloesol	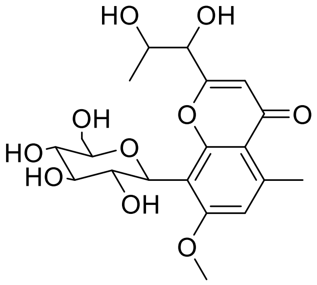	410.1577
42	*Aloe*	7-O-methylaloesin	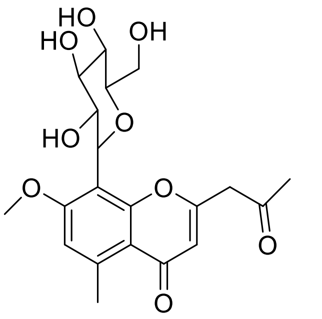	408.142

43	*Aloe*	Aloeresin B	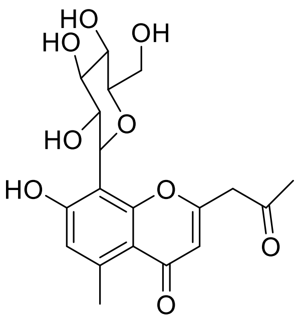	394.1264
44	*Aloe*	7-O-methylaloeresin A	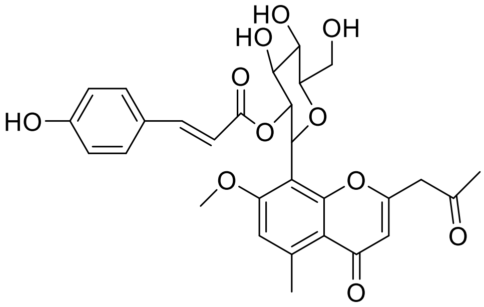	554.1788
45	*Aloe*	Aloeresin F	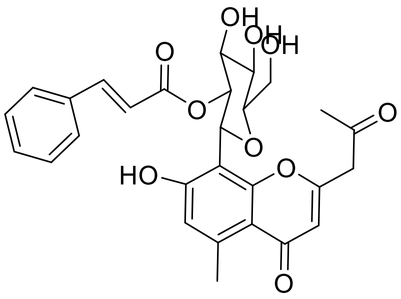	524.1683
46	*Aloe*	(E)-2-acetonyl-8-(2'-O-caffeoyl)-β-D-glucopyranosyl-7-methoxy-5-methylchromone	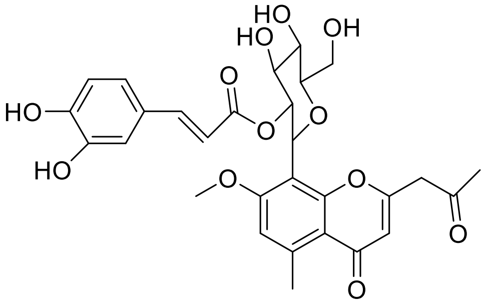	570.1737
47	*Aloe*	(E)-2-acetonyl-8-(2',6'-di-O,O-coumaroyl)-β-D-glucopyranosyl-7-hydroxy-5-methylchromone	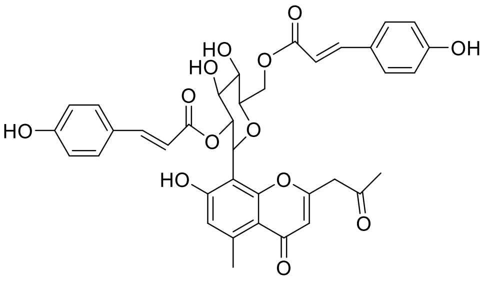	686.1999

48	*Aloe*	Aloeresin G	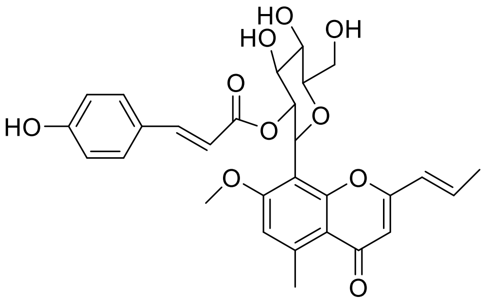	538.1839
49	*Aloe*	(E)-2-acetonyl-8-(2'-O-feruloyl)-β-D-glucopyranosyl-7-methoxy-5-methylchromone	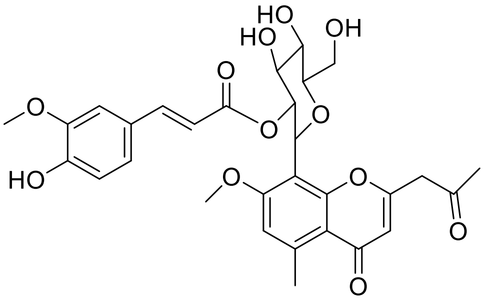	584.1894
50	*Aloe*	7-hydroxy-2,5-dimethylchromone	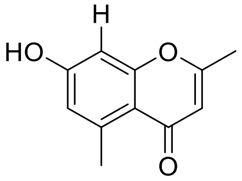	190.063
51	*Aloe*	2-acetonyl-8-(2'-O-cinnamoyl)-β-D-glucopyranosyl-5-methylchromone	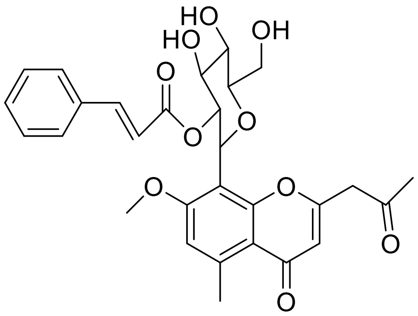	686.2211
52	*Aloe*	Aloenin	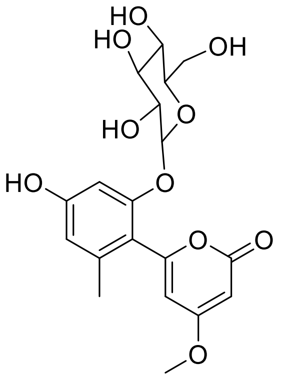	410.1213

53	*Aloe*	5-(2'-oxo-4'-hydroxypentyl)-2-(β-glucopyranosyl-oxy-methyl)chromone	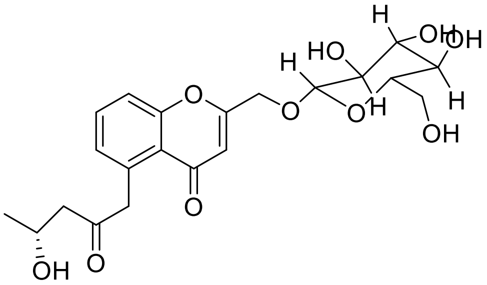	438.1526
54	*Aloe*	5-((S)-2'-oxo-4'-hydroxypentyl)-2-methoxychromone	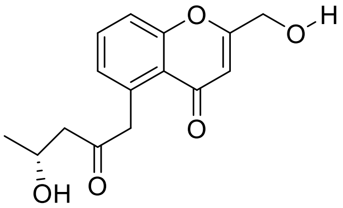	276.0998
55	*Aloe*	Plicataloside	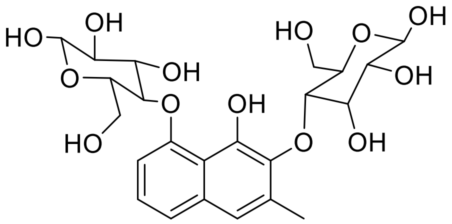	514.1686
56	*Aloe*	Aloveroside A	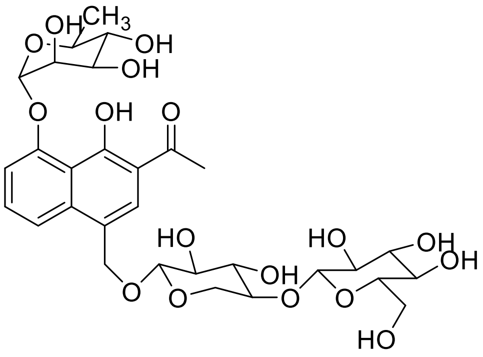	672.2266
57	*Aloe*	Aloveroside B	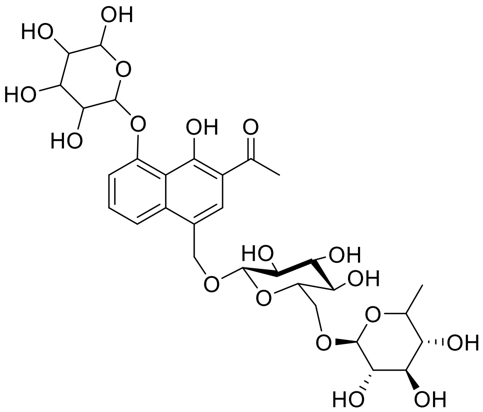	688.2215

58	*Aloe*	8-(α-L-rhamnopyranosyloxy)-3-(β-D-xylopyranosyl oxymethyl)naphthalen-ol	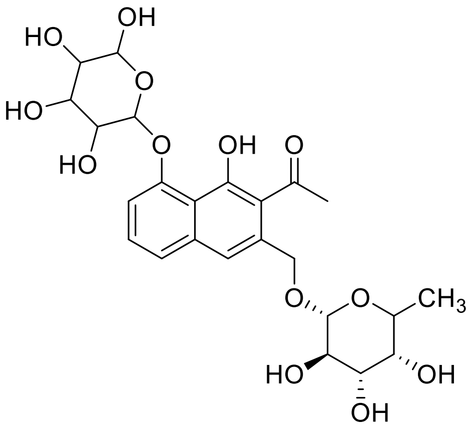	526.1686
59	*Aloe*	Elgonica-dimer A	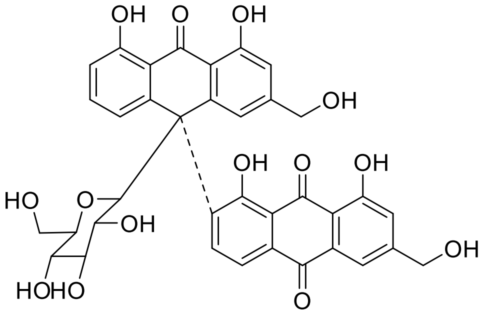	686.1636
60	*Aloe*	p-Coumaroylaloenin	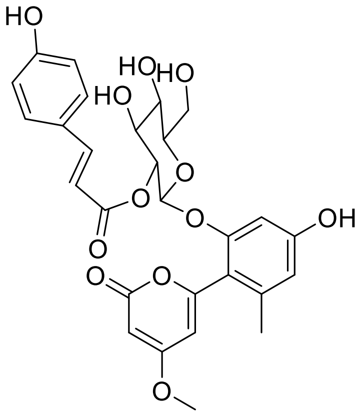	556.1581
61	*Aloe*	Aloeresin D	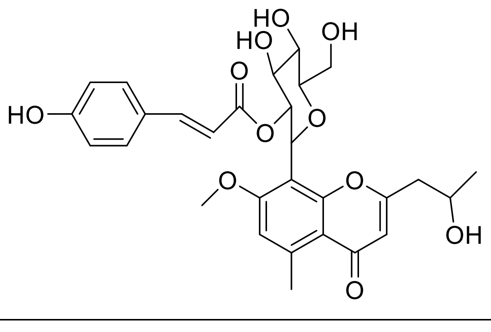	556.1945

62	*Aloe*	Rabaichromone	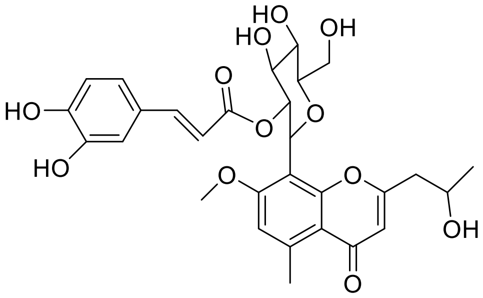	572.1894
63	*Aloe*	4'-O-glucosylisoaloeresin D Ⅰ	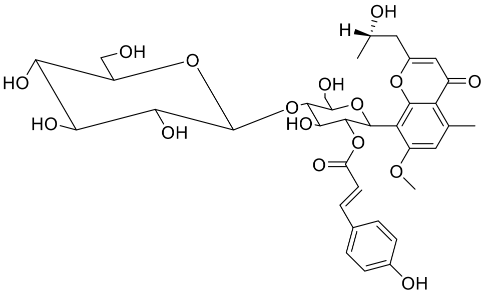	718.2473
64	*Aloe*	Aloeresin A	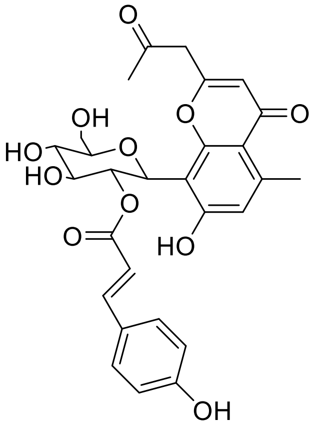	540.1632
65	*Aloe*	Aloeresin H	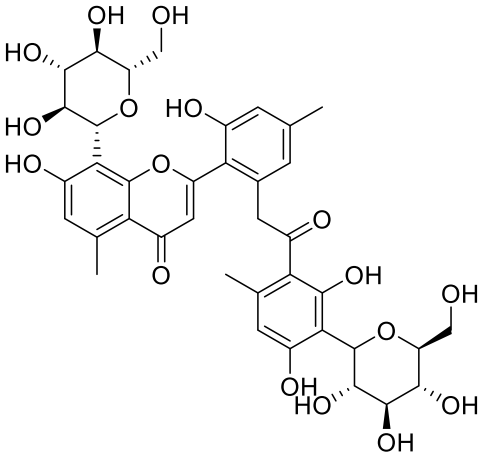	770.2422

66	*Aloe*	Littoraloside	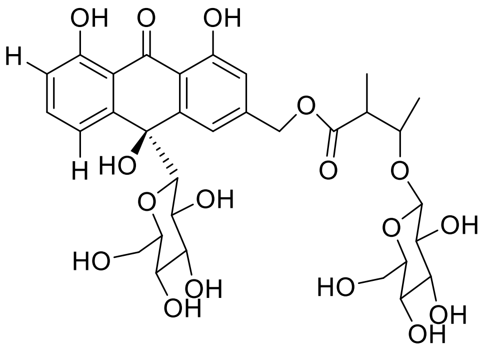	696.2266
67	*Aloe*	6'-malonylnataloin	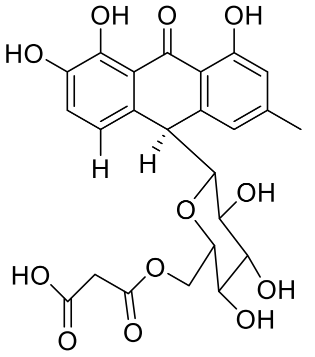	504.1268
68	*Aloe*	8-C-glucosyl-7-O-methyl-aloediol	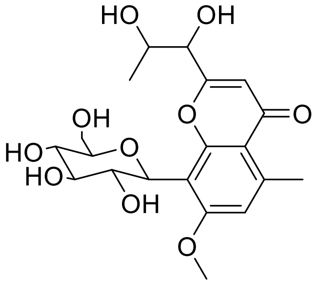	426.1526
69	*Aloe*	Aloeresin C	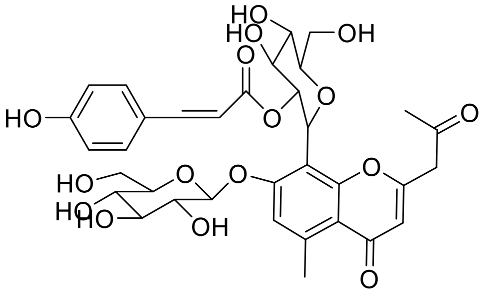	702.216
70	*Aloe*	Aloesone	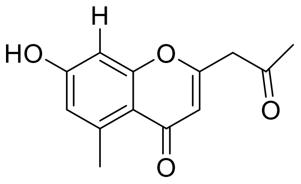	232.0736

71	*Aloe*	2-acetonyl-7-hydroxy-8-(3-hydroxyactonyl)-5-methylchromone	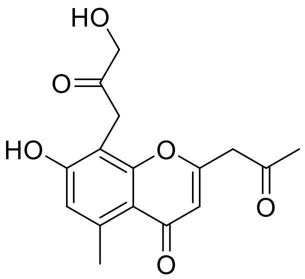	304.0947
72	*Aloe*	2-acetonyl-8-(2-furoylmethyl)-7-hydroxy-5-methylchromone	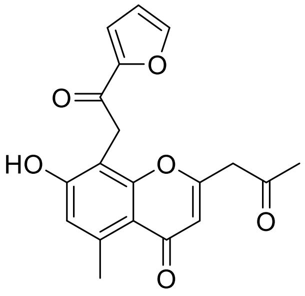	340.0947
73	*Aloe*	Feroxin A	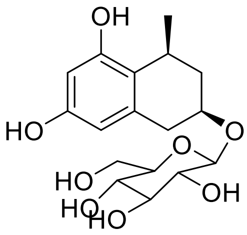	356.1471
74	*Aloe*	Feroxin B	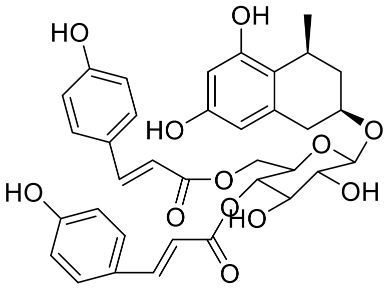	648.2207
75	*Aloe*	Feroxidin	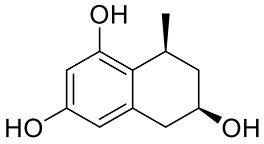	194.0943

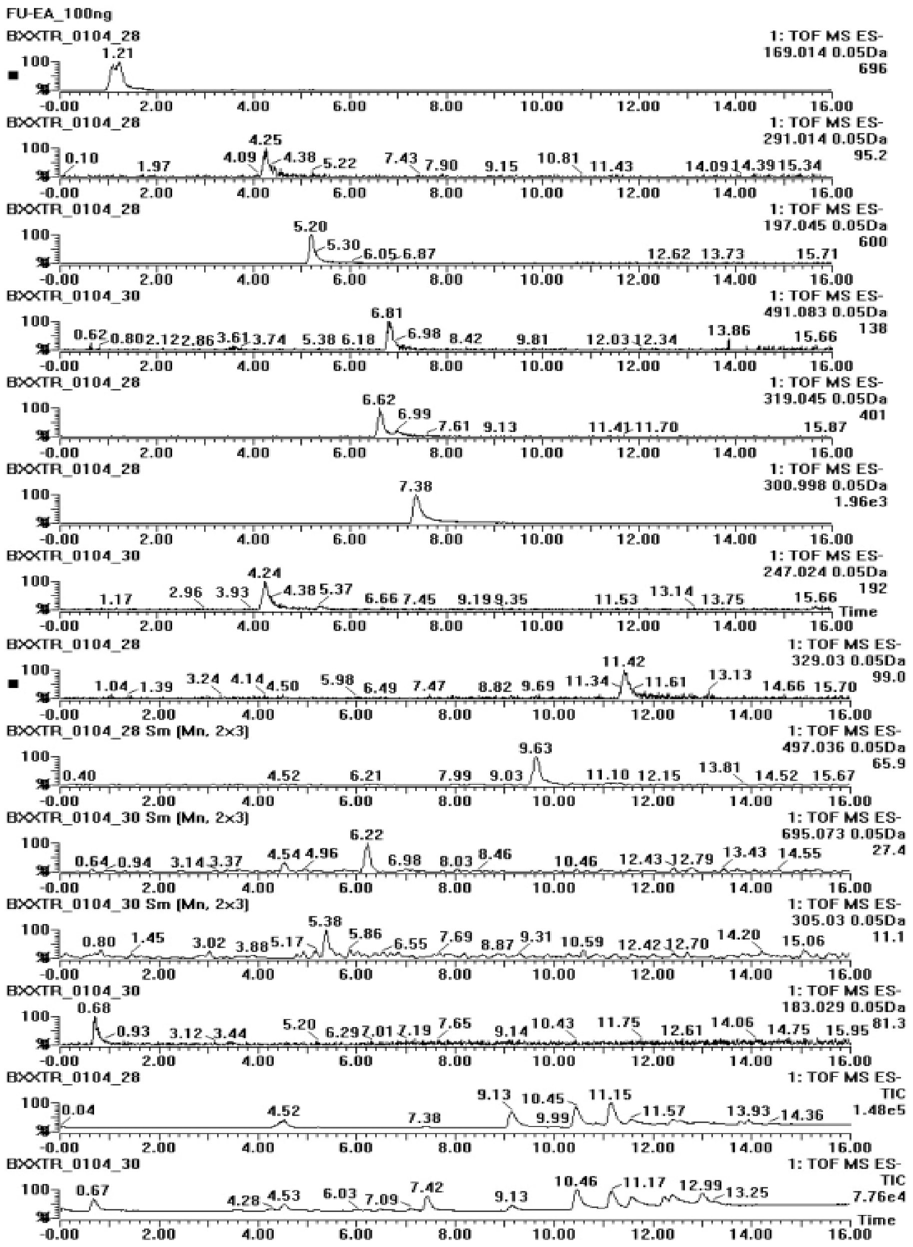

Fig.S3 Total ion chromatogram of extracts of BXXTR and extracted ion chromatograms of compounds obtained from UPLC-TOF-MS analysis.

From up to down: Gallic acid; Brevifolin carboxylic acid; Ethyl gallate; 3,3’-2-di-O-methyl ellagic acid-4-O-β-D-glucopyranoside; Ethyl brevifolin carboxylate; Ellagic acid; Brevifolin; 3,3'-di-O-methylellagic acid; 7’-ethyl-sanguisorbic acid dilactone; Furosin; Methyl brevifolin carboxylate; Methyl gallate. BXXTR-0104-28 is the total ion chromatogram of the Ethyl acetate extraction layer tested in ES- mode; BXXTR-0104-30 is the total ion chromatogram of the n-Butanol extraction layer tested in ES- mode. The Exact Mass error of all compounds does not exceed ±5ppm. (TIF)

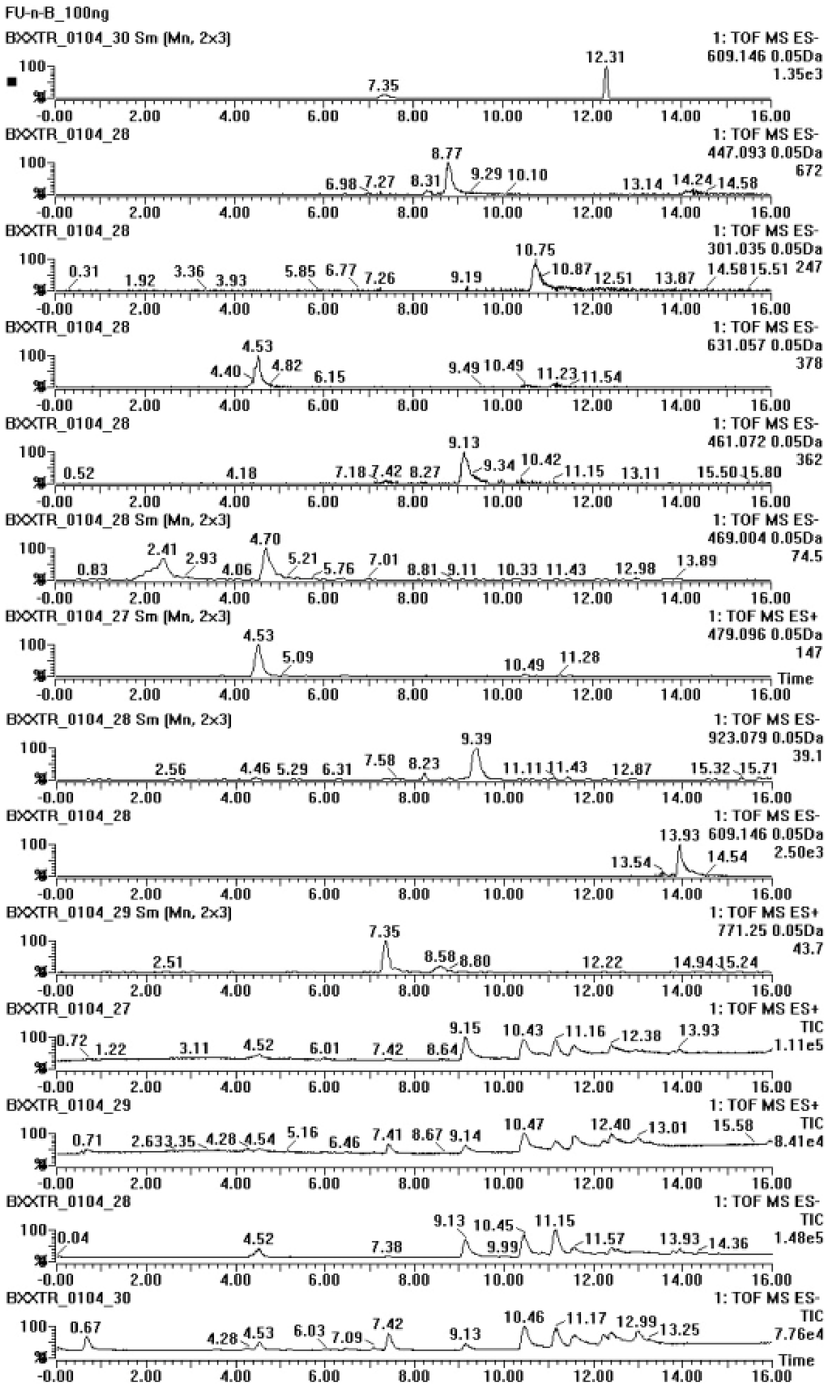

Fig.S4 Total ion chromatogram of extracts of BXXTR and extracted ion chromatograms of compounds obtained from UPLC-TOF-MS analysis.

From up to down: Apigenin-7-O-β-D-apiofuranosyl(1-2)-β-D-glucopyranoside; Luteolin-7-O-β-D-glucopyranoside; Quercetin; Terflavin D; 3,3'-di-O-methyl-4-O-(β-D-xylopyranosyl)ellagic acid; (S)-Flavogallonic acid; Triethyl chebulate; Euphormisin M2; Rutin; Aloeresin H. BXXTR-0104-27 is the total ion chromatogram of the Ethyl acetate extraction layer tested in ES+ mode; BXXTR-0104-28

is the total ion chromatogram of the Ethyl acetate extraction layer tested in ES- mode; BXXTR-0104-29 is the total ion chromatogram of the n-Butanol extraction layer tested in ES+ mode; BXXTR-0104-30 is the total ion chromatogram of the n-Butanol extraction layer tested in ES- mode. The Exact Mass error of all compounds does not exceed ±5ppm.

(TIF)

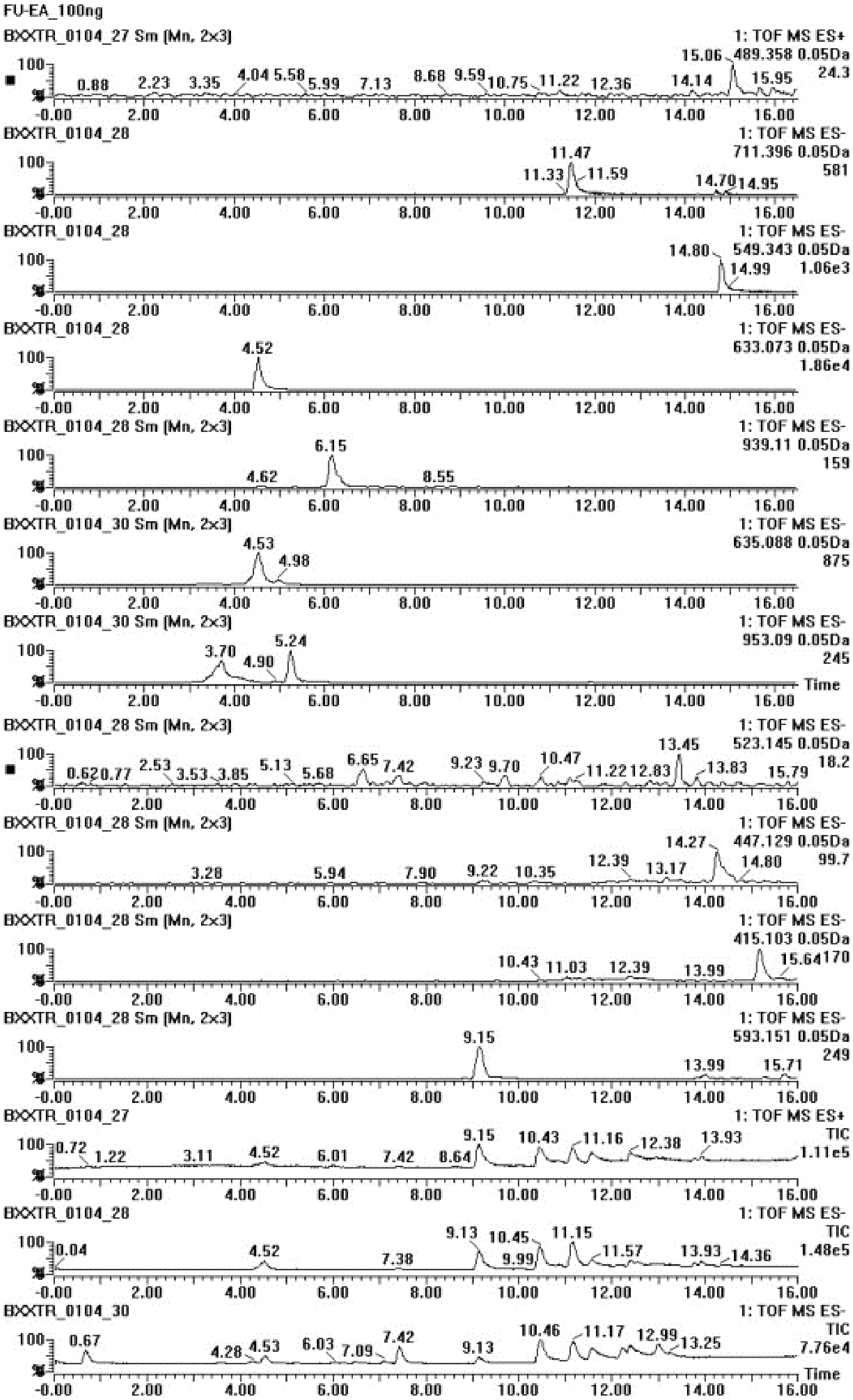

Fig.S5 Total ion chromatogram of extracts of BXXTR and extracted ion chromatograms of compounds obtained from UPLC-TOF-MS analysis.

From up to down: Arjunolic acid; Chebuloside Ⅱ; Arjugenin; Corilagin; 1,2,3,4,6-penta-O-galloyl-β-D-glucose; 1,3,6-tri-O-galloyl-β-D-glucose; Chebulagic acid; Aloesaponol Ⅰ-6-O-β-D-glucoside; Aloenoside; Chrysophanol glucoside; Aloe-emodin diglucoside. BXXTR-0104-27 is the total ion chromatogram of the Ethyl acetate extraction

layer tested in ES+ mode; BXXTR-0104-28 is the total ion chromatogram of the Ethyl acetate extraction layer tested in ES- mode; BXXTR-0104-30 is the total ion chromatogram of the n-Butanol extraction layer tested in ES- mode. The Exact Mass error of all compounds does not exceed ±5ppm. (TIF)

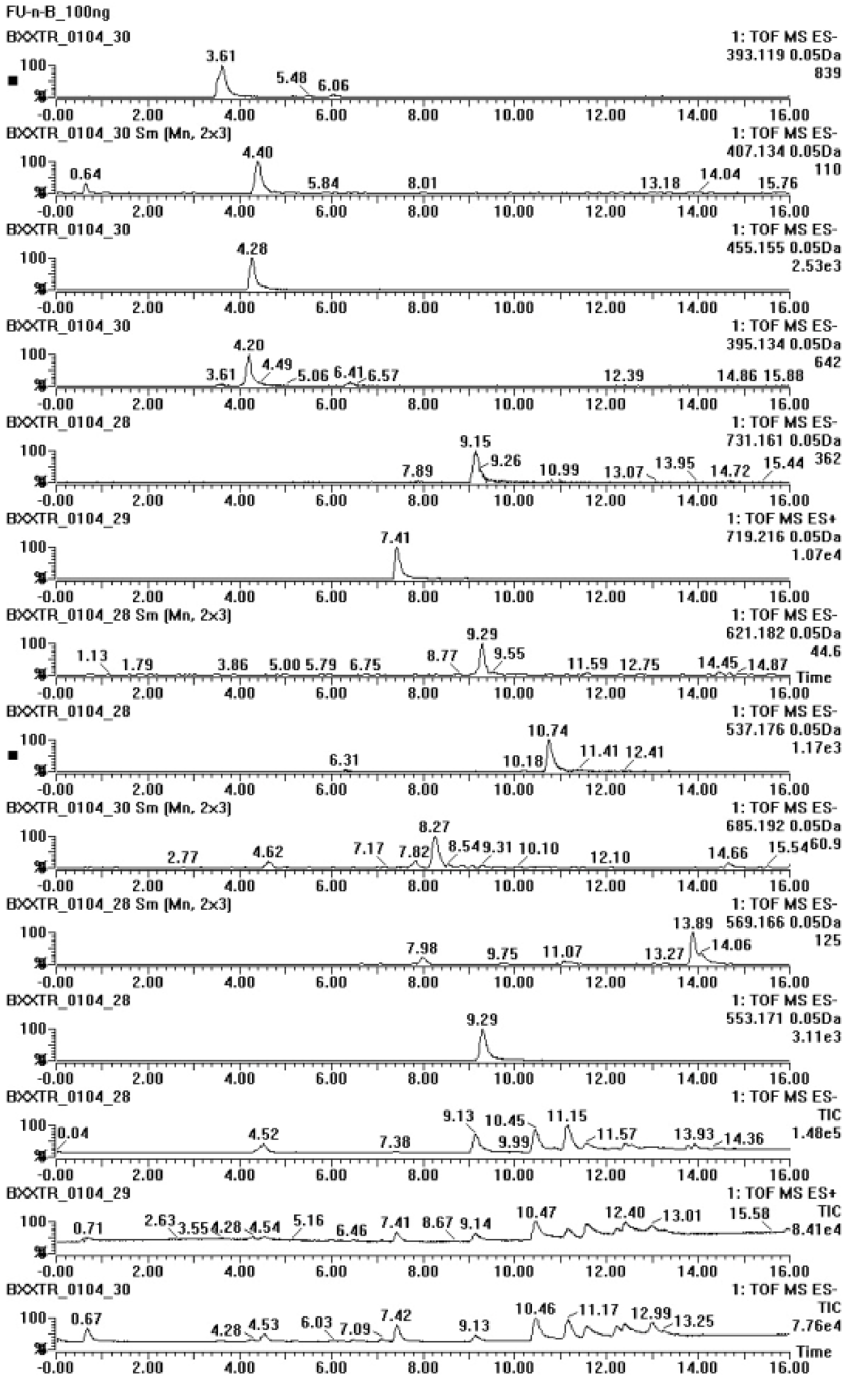

Fig.S6 Total ion chromatogram of extracts of BXXTR and extracted ion chromatograms of compounds obtained from UPLC-TOF-MS analysis.

From up to down: Aloeresin B; 7-O-methylaloesin; 8-C-glucosyl-7-O-methyl-(S)-aloesol; 8-C-glucosyl-(S)-aloesol; Elgonica-dimer A; Littoraloside; Littoraloin; Aloeresin G; (E)-2-aceton-

yl-8-(2',6'-di-O,O-coumaroyl)-β-D-glucopyranosyl-7-hydroxy-5-methylchromone; aloeresin F; 7-O-methyl-aloeresin A. BXXTR-0104-28 is the total ion chromatogram of the Ethyl

acetate extraction layer tested in ES- mode; BXXTR-0104-29 is the total ion chromatogram of the n-Butanol extraction layer tested in ES+ mode; BXXTR-0104-30 is the total ion chromatogram of the n-Butanol extraction layer tested in ES- mode. The Exact Mass error of all compounds does not exceed ±5ppm. (TIF)

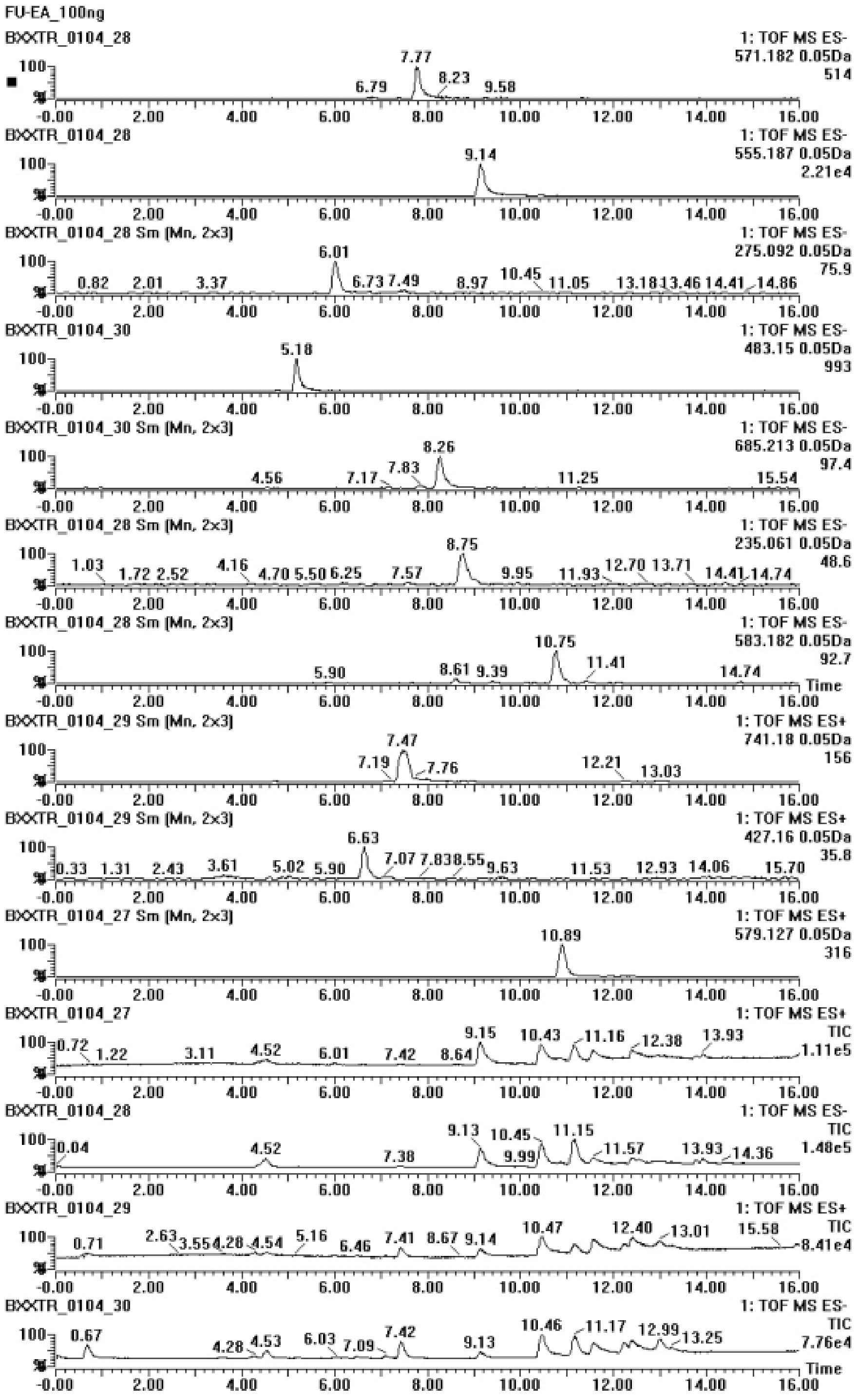

Fig.S7 Total ion chromatogram of extracts of BXXTR and extracted ion chromatograms of compounds obtained from UPLC-TOF-MS analysis.

From up to down: Rabaichromone; aloeresin D; 5-((S)-2'-oxo-4'-hydroxypentyl)-2-methoxychro-mone; 5-(2'-oxo-4'-hydroxypentyl)-2-(β-glucopyranosyl-oxy-methyl)chromone; 2-acetonyl-8-(2'-O-cinnamoyl)-β-D-glucopyranosyl-5-methylchromone;

7-hydroxy-2,5-dimethylchromone;

(E)-2-acetonyl-8-(2'-O-feruloyl)-β-D-glucopyranosyl-7-methoxy-5-methylchromone; aloeresin C; 8-C-glucosyl-7-O-methyl-aloediol; aloeresin A. BXXTR-0104-27 is the total ion chromatogram of the Ethyl acetate extraction layer tested in ES+ mode; BXXTR-0104-28 is the total ion chromatogram of the Ethyl acetate extraction layer tested in ES- mode; BXXTR-0104-29 is the total ion chromatogram of the n-Butanol extraction layer tested in ES+ mode; BXXTR-0104-30 is the total ion chromatogram of the n-Butanol extraction layer tested in ES- mode. The Exact Mass error of all compounds does not exceed ±5ppm. (TIF)

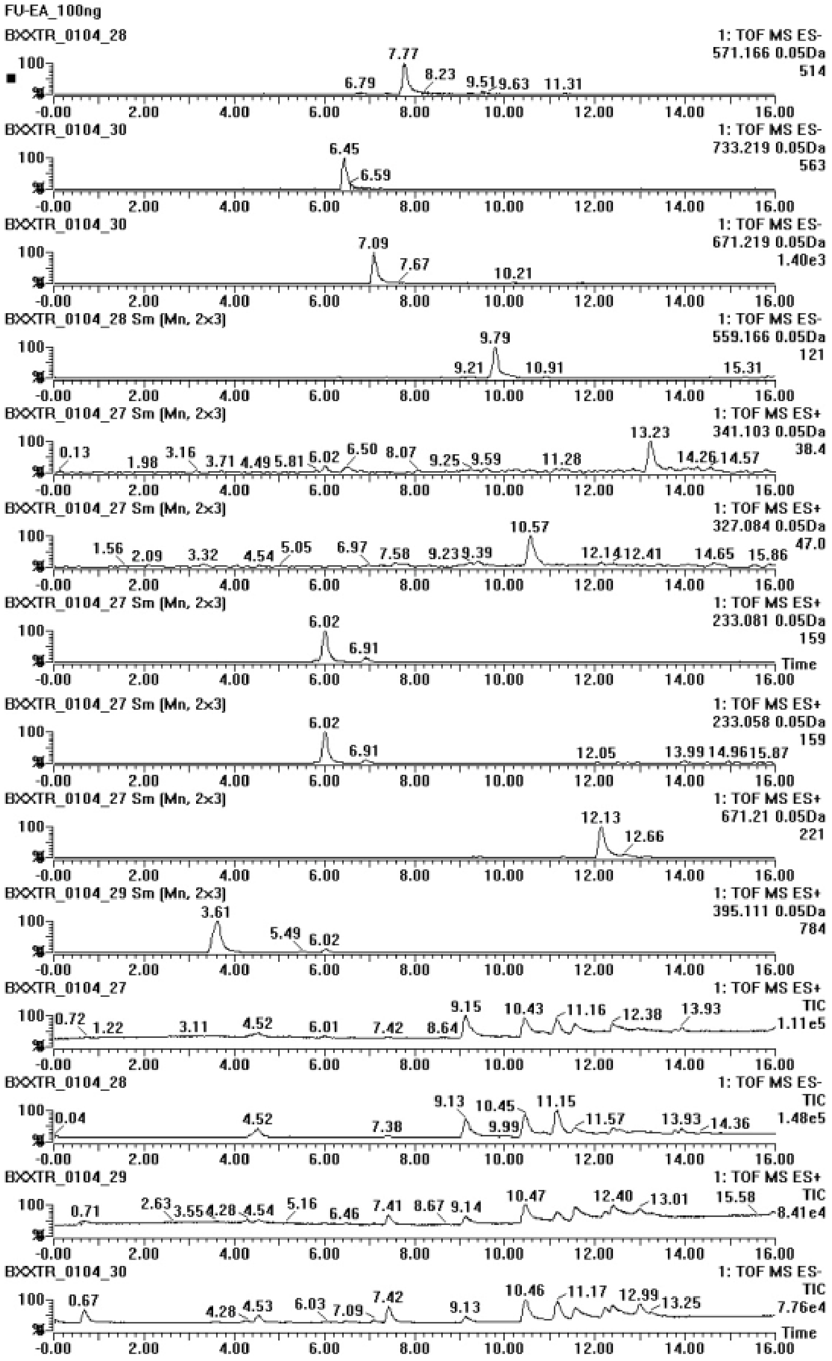

Fig.S8 Total ion chromatogram of extracts of BXXTR and extracted ion chromatograms of compounds obtained from UPLC-TOF-MS analysis.

From up to down: 8-(α-L-rhamnopyranosyloxy)-3-(β-D-xylopyranosyl oxymethyl)naphthalenol; Aloveroside B; Aloveroside A; Plicataloside; 2-acetonyl-8-(2-furoylmethyl)-7-hydroxy-5-methyl-Chromone; 2-acetonyl-7-hydroxy-8-(3-hydroxyactonyl)-5-methylchromone; Aloesone; Feroxidin; Feroxin B; Feroxin A. BXXTR-0104-27 is the total ion chromatogram of the Ethyl acetate extraction layer tested in ES+ mode; BXXTR-0104-28 is the total ion chromatogram of the

Ethyl acetate extraction layer tested in ES- mode; BXXTR-0104-29 is the total ion chromatogram of the n-Butanol extraction layer tested in ES+ mode; BXXTR-0104-30 is the total ion chromatogram of the n-Butanol extraction layer tested in ES- mode. The Exact Mass error of all compounds does not exceed ±5ppm. (TIF)

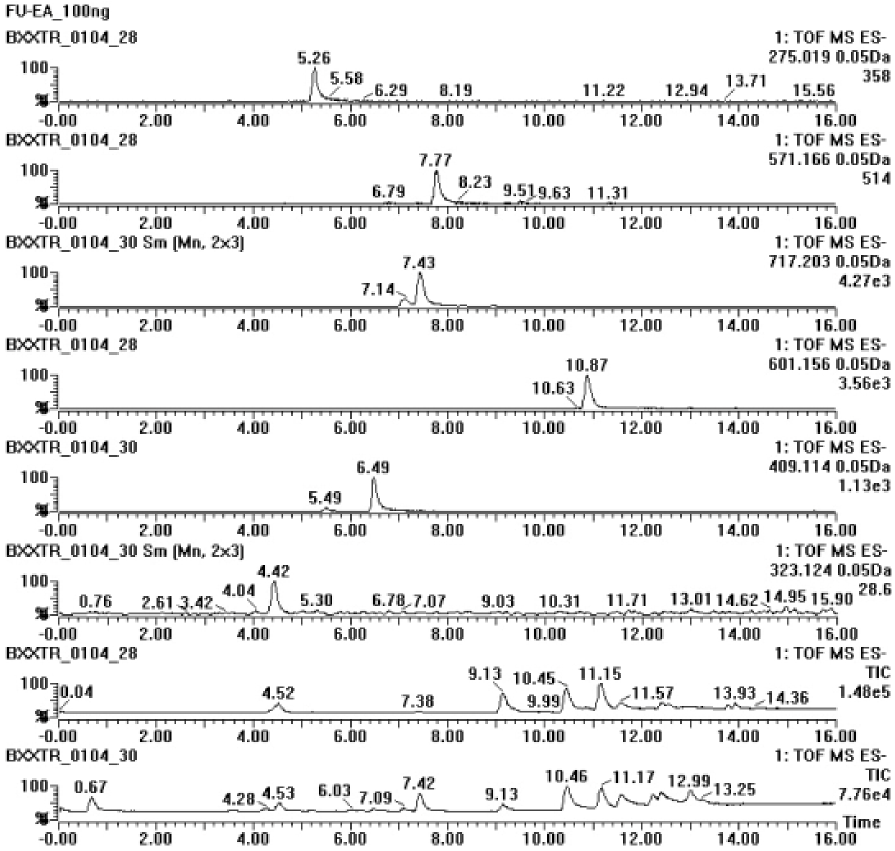

Fig.S9 Total ion chromatogram of extracts of BXXTR and extracted ion chromatograms of compounds obtained from UPLC-TOF-MS analysis.

From up to down: 3,4,8,9,10-pentahydroxydibenzo-[β,D]pyran-6-one; 10-O-β-D-glucopyranosyl aloenin; aloenin B; p-Coumaroylaloenin; Aloenin; 1-(2'3'4'5'-tetrahydroxypentyl)-6,7-dimethyl-

quinoxaline-2,3-(1H,4H)-dione. BXXTR-0104-28 is the total ion chromatogram of the Ethyl acetate extraction layer tested in ES- mode; BXXTR-0104-30 is the total ion chromatogram of the n-Butanol extraction layer tested in ES- mode. The Exact Mass error of all compounds does not exceed ±5ppm. (TIF)

Table S2. The genes change IMQ *vs* control group ($p < 0.05$ and fold change > 2)

Table S3. The genes change IMQ-BXXTR group *vs* IMQ group ($p < 0.05$ and fold change > 2)
